# Antimony-modified porous lamellar zinc as reversible and stable anode for high-performance alkaline aqueous zinc–air battery

**DOI:** 10.1080/14686996.2026.2658327

**Published:** 2026-05-07

**Authors:** Muhammad Afiq Irfan Mohd Shumiri, Hikari Sakaebe, Abdillah Sani Mohd Najib, Nor Akmal Fadil

**Affiliations:** a Materials Research and Consultancy Group, Faculty of Mechanical Engineering, Universiti Teknologi Malaysia, Johor Bahru, Malaysia; b Institute for Materials Chemistry and Engineering, Kyushu University, Kasuga-koen, Kasuga, Japan; c Department of Materials, Manufacturing and Industry, Faculty of Mechanical Engineering, Universiti Teknologi Malaysia, Johor Bahru, Malaysia

**Keywords:** metal-air batteries, Zn anode, dendrite-free, surface modification, structural design, cycle stability

## Abstract

Monometallic Zn is a promising anode material for post-lithium batteries owing to its high theoretical capacity, low cost and natural abundance. However, the practical application in zinc–air batteries (ZABs) remain limited by uncontrollable dendrite growth and severe voltage polarization during repeated cycling. Herein, we report a self-supported Sb-modified porous lamellar zincophilic electrode (Sb-pZn) as a reversible, dendrite-free and high-performance hostless Zn anode for aqueous alkaline rechargeable ZABs. The porous lamellar framework is fabricated by selectively dissolving the Al component from the eutectic Zn–Al alloy via chemical dealloying, followed by surface modification through galvanic replacement in SbCl_3_ solution. The Sb-pZn anode with lamellar thickness of ~2920 nm (Sb-pZn-2920) enables continuous electron and ion transport pathways through quasi-periodic lamellar channels and interconnected ligament network, ensuring uniform Zn deposition and dissolution. Moreover, Sb incorporation introduces abundant zincophilic sites that accelerate charge-transfer kinetics and enhance reversibility. As a result, the Sb-pZn-2920 anode shows exceptional Zn plating-stripping behaviours with ultralow overpotential of 31.7 mV for up to 1700 h at 2 mA cm^−2^ in symmetric cells and maintains stable charge–discharge cycling for 240 cycles at 20 mA cm^−2^ under ambient conditions in ZAB full cells. This work presents a facile and scalable strategy for constructing high-performance Zn anodes and offers valuable insights into the development of practical, durable and reversible ZABs.

## IMPACT STATEMENT

This work presents a novel strategy for designing high-performance zinc anodes, providing key insights into achieving highly reversible, stable and practical zinc-air batteries for next-generation energy storage.

## GRAPHICAL ABSTRACT

**Figure:**
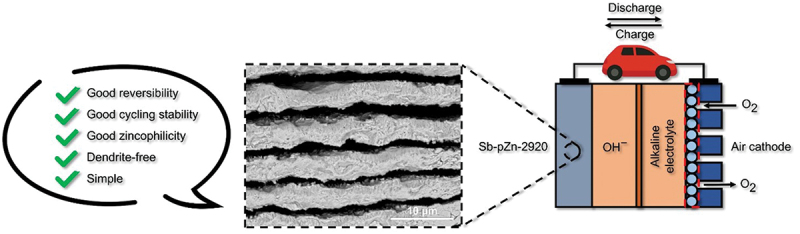

## Introduction

Due to the widespread adoption of electric vehicles, there is urgent demand for highly reliable and cost-effective rechargeable batteries. Currently, lithium-ion batteries (LIBs) dominate nearly all commercial electric vehicle models [1,2]. However, their performance has approached theoretical limits, raising concerns about further advancement [3–5]. Moreover, LIBs face sustainability challenges including high production costs, safety risks associated with flammable electrolytes and the scarcity of lithium resources [4,6]. These limitations underscore the need for developing alternative energy storage systems. Among various electrochemical energy storage technologies, aqueous alkaline zinc–air batteries (ZABs) have attracted growing attention. ZABs combine the advantages of traditional Zn-based batteries and fuel cells, offering energy density four times higher than LIBs [7–9]. Metallic Zn possesses high volumetric and gravimetric capacities (5854 mAh cm^−3^ and 820 mAh g^−1^, respectively), low Zn/Zn^2+^ redox potential (−0.76 V versus the standard hydrogen electrode), natural abundance and low material cost [10–12]. In addition, aqueous alkaline electrolytes provide high ionic conductivity (up to 1 S cm^−1^), while the two-electron Zn/Zn^2+^ redox reaction enables excellent rate capability [13,14]. The air cathode utilizes oxygen from ambient air as a freely available reactant, which further reduces battery weight and enhances energy efficiency [15,16]. Collectively, these features make ZABs highly attractive candidates for safe, low-cost and high-power energy storage, especially for electric vehicle applications.

The recent resurgence of interest in ZABs has been largely driven by advances in air cathode design, including the development of bifunctional oxygen catalysts based on manganese oxides, vanadium oxides and nickel hydroxide [17]. Despite these achievements, the reversibility of monometallic Zn anodes remains a major bottleneck, limiting the long-term stability of ZABs. Conventional planar Zn anodes suffer from dendrite formation, corrosion, hydrogen evolution and by-product accumulation during repeated stripping-plating cycles, leading to severe voltage polarization, low coulombic efficiency and rapid capacity fading [18–20]. Since the active Zn material on the anode surface is directly involved and consumed during redox reactions, anode design and modification are critical to achieve durable ZABs. Various strategies have been explored including electrolyte engineering with additives to regulate Zn^2+^ solvation-desolvation and local electric field distribution [21–23], alloying-induced surface nanostructuring to guide uniform Zn deposition [24,25] and the construction of protective or artificial solid electrolyte interphase layers to suppress side reactions [26–28]. However, bulk monometallic Zn anodes still suffer from large voltage polarization, particularly at high current densities which promotes dendrite growth during prolonged cycling, thereby compromising the stability of open-system ZABs. Designing porous Zn structures is a straightforward approach to mitigate these issues by providing high surface area to reduce local current density and enhance Zn^2+^ transport at the electrode–electrolyte interface [29,30]. Nevertheless, randomly distributed pores and excessive porosity increase surface chemical activity due to abundant low-coordination atoms, leading to severe side reactions. Carbon-based materials, such as graphene [31,32], carbon nanotubes [33] and metal–organic framework derivatives [34] have also been investigated as zinc-free anodes because their low lattice mismatch can promote Zn nucleation. However, they typically require metallic substrates for support, and their stacked low-dimensional structures reduce accessible nucleation sites while introducing additional interfaces with high contact resistance that impede electron transport and limit efficient Zn electrodeposition [35]. In this regard, current progress on anode modification strategies remains limited. Therefore, it is highly desirable to explore novel Zn-based anode materials that can circumvent these irreversibility issues for constructing high-performance ZABs.

Herein, we report self-supported Sb-modified porous lamellar zincophilic electrode (Sb-pZn) as a reversible, dendrite-free and high-performance hostless Zn anode for aqueous alkaline rechargeable ZABs. Benefiting from the tunable lamellar spacing of the eutectic Zn–5Al (wt%) precursor alloy formed under controlled solidification, the Sb-pZn anode with lamellar thickness of ~2920 nm (Sb-pZn-2920) exhibits superior electrochemical performance. The quasi-periodic lamellar channels and interconnected ligament network enable continuous electron and ion transport pathways, ensuring uniform reaction kinetics during Zn plating and stripping. The incorporation of Sb introduces highly zincophilic sites that guide homogeneous Zn deposition and enhance electrochemical reversibility. In a strong alkaline KOH–ZnO electrolyte, the Sb-pZn-2920 anode demonstrates markedly improved Zn nucleation and deposition kinetics. In symmetric cells, Sb-pZn-2920 shows highly stable and reversible Zn plating-stripping with an ultralow overpotential of 31.7 mV maintained up to 1700 h at a current density of 2 mA cm^−2^. When assembled into ZAB full cell, the Sb-pZn-2920||O_2_ delivers high discharge capacity of 580.7 mAh g^−1^ at 20 mA cm^−2^, indicating excellent anode utilization. Moreover, the Sb-pZn-2920||O_2_ displays outstanding rate capability and cycling durability, maintaining stable charge–discharge profile up to 240 continuous cycles at 20 mA cm^−2^ under ambient conditions. This work presents a simple and scalable strategy for constructing high-performance Zn anodes and offers valuable insights toward the development of next-generation aqueous ZABs with enhanced reversibility, stability and practical applicability.

## Experimental section

### Preparation of Sb-modified porous lamellar Zn anode

Precursor alloy of eutectic Zn–5Al (wt%) was first produced by induction melting of high-purity Zn (99.994%) and Al (99.996%) in alumina crucible. The alloy ingot was obtained through pouring casting, of which the solidification process was controlled by making use of different cooling techniques by water-, air- and furnace-cooled method [36]. After being cut into sheets with a thickness of 0.5 mm using a precision cutter, the as-cast precursor alloy was chemically dealloyed for 4 h at 50°C to prepare porous lamellar Zn (pZn), wherein the less noble α-Al component was selectively dissolved in 4 M NaOH solution. After rinsing with pure water and ethanol several times, the dealloyed pZn was immersed in 0.15 M SbCl_3_ ethanol solution under stirring for 3 min at room temperature to obtain Sb-modified pZn (Sb-pZn) via galvanic replacement reaction. The as-prepared Sb-pZn was further washed with pure water to remove residual chemicals and directly used as the anode in symmetric and full cells.

### Preparation of air cathode

Air cathode was prepared with catalyst composed of manganese dioxide (MnO_2_) and nickel(II) hydroxide (Ni(OH)_2_) as bifunctional electrocatalysts for the oxygen reduction (ORR) and oxygen evolution (OER) reactions, respectively. MnO_2_, Ni(OH)_2_, commercial carbon black and polytetrafluoroethylene (PTFE) were gently mixed in agate mortar in weight ratio of 3:3:3:1 and dispersed into ethanol and water at volume ratio of 1:1. Carbon black was used to enhance the electrical conductivity, while PTFE acted as a binder to form the catalytic layer. The solution was stirred for 10 min, followed by 10 min of sonication. The catalyst ink was then emulsified for 1 min at 30,000 rpm using high-shear homogenizer to ensure uniform dispersion and finally spread onto commercial PTFE-treated Toray carbon paper using the doctor blade method until the desired catalyst loading of 5 mg cm^−2^ was achieved.

### Material characterization

The electron micrographic structures were characterized by using scanning electron microscope (JEOL NeoScope Benchtop SEM JCM-7000, Japan) equipped with energy-dispersive X-ray spectrometry. X-ray diffraction (XRD) measurements were carried out on Rigaku Mini Flex 600 (Japan) diffractometer with Cu *K*α radiation (*λ* = 0.15418 nm). X-ray photoelectron spectroscopy (XPS) analysis was conducted on ULVAC–PHI Quantes (Japan) with monochromatic Al *K*α source (1486.6 eV). Charging effect was compensated by referencing the binding energy to the adventitious C 1s peak (284.8 eV). The 3D surface morphology of Sb-pZn was analysed using atomic force microscopy (AFM, Bruker Multimode 8, Germany) operating in tapping mode with scan size of 20 µm in both *X* and *Y* directions and *Z*-axis range of 15 µm. The contact angle was measured using a self-assembled setup equipped with digital camera, backlight and adjustable sample stage.

### Electrochemical measurement

The electrochemical performance was tested using electrochemical workstation (Biologic SP-300, French and Gamry 1010b, USA) and battery test (Hokuto Denko, 210 HJ1020MSD8, Japan). Symmetric cells were assembled in HS cells from Hohsen Corp., Japan under ambient air using two identical Sb-pZn, pZn and bare Zn sheets (15 mm diameter, 0.5 mm thickness), separated by Whatman glass fiber membrane (20 mm diameter, 100 µm thick, 75% porosity) in 200 µL of 8.5 M KOH and 0.6 M ZnO aqueous electrolyte. Electrochemical Zn stripping-plating behaviour was measured at varied current densities from 1 to 20 mA cm^−2^. CV measurements of symmetric cells were conducted at scan rate of 10 mV/s, with the potential swept from the open-circuit voltage (OCV) over a symmetric voltage interval of −1.0 to 1.0 V (versus Zn/Zn^2+^). EIS measurements were taken over the frequency range of 10 kHz to 0.1 Hz with an amplitude of 10 mV at varied temperatures from 15 to 40°C. Chronopotentiometry measurements for nucleation overpotentials were performed at varied current densities from 1 to 5 mA cm^−2^. Cycling durability tests were conducted at 2 and 10 mA cm^−2^. Tafel curves were tested in the three-electrode system using graphite as the counter electrode and Ag/AgCl as the reference electrode, with a scan rate of 5 mV s^−1^ over a potential range of −0.25 V to +0.25 V versus OCV. For full cell test, custom ZABs were assembled under ambient air using the as-prepared anode paired with the air cathode. The polarization test was conducted under galvanodynamic mode with a scan rate of 0.1 mA s^−1^. The self-discharge behaviour was evaluated by measuring the OCV over a continuous period of 20 h under ambient conditions. CV measurements of ZAB full cells were performed in the voltage range of 0.2 to 2.2 V at a scan rate of 1 mV s^−1^. Galvanostatic charge–discharge tests were conducted at current densities ranging from 10 to 50 mA cm^−2^. Cycling tests were performed at 10 and 20 mA cm^−2^ and terminated when the voltage reached 0 V during discharge or 2.5 V during charge.

## Result and discussion

### Characterizations of Sb-pZn

**Figure 1.**
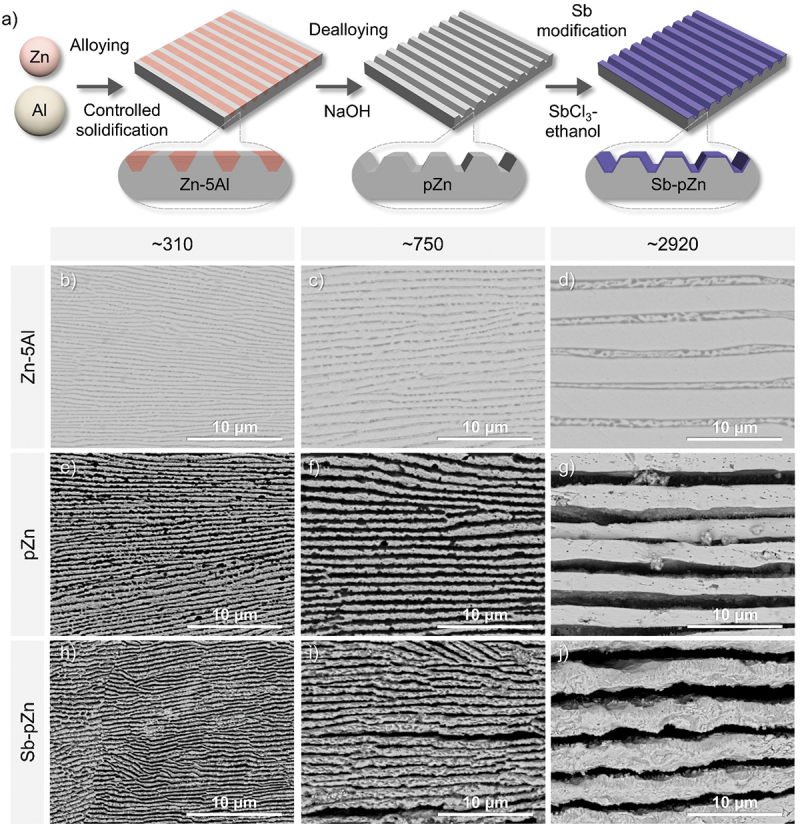
Preparation and microstructural properties of the modified Zn-based anode. (a) Schematic illustration of Sb-pZn fabricated by alloying-dealloying technique and surface modification with Sb. (b–d) SEM images of eutectic Zn–5Al (wt%) precursor alloy prepared by induction melting of high-purity Zn and Al. (e–g) SEM images of pZn prepared by chemical dealloying in NaOH solution. (h–j) SEM images of Sb-pZn showing incorporation of Sb on the porous lamellar Zn by galvanic replacement reaction.

Figure 1a schematically illustrates the preparation process of Sb-pZn anode. Eutectic Zn–5Al (wt%) precursor alloy was initially fabricated through a simple and scalable metallurgical process involving the alloying of pure Zn and Al, followed by casting under controlled solidification technique using water-, air- and furnace-cooled methods. Figure S1 illustrates the XRD patterns of eutectic Zn–5Al precursor alloy, with dominant peaks corresponding to the hexagonal close-packed (hcp) β-Zn phase (JCPDS 04–0831) and minor peaks associated with the face-centered cubic (fcc) α-Al phase (JCPDS 04–0787). The solidification process strongly influences the final-phase distribution, as the growth of solid phases depends on atomic diffusion [37,38]. Water cooling induces rapid solidification rate, resulting in the formation of fine Zn lamellae with an average thickness of ~310 nm (Figure 1b), as the fast cooling rate limits α-Al diffusion and promotes localized precipitation of the α-Al phase. Under air cooling, Zn lamellae exhibit moderate coarsening, reaching thickness of ~750 nm (Figure 1c). In contrast, furnace cooling produce significantly coarser Zn lamellae with thickness of ~2920 nm (Figure 1d) owing to the slow cooling rate, which facilitates extensive atomic diffusion and lamellar growth. This tunable lamellar pattern offers precise structural control over the precursor alloy, thereby enabling the rational design of the resultant porous framework.

Subsequently, the self-supported porous Zn (pZn) was prepared through a straightforward chemical dealloying process as reported in our previous work [36]. The pZn sheet was obtained by selectively etching the less noble Al component from the eutectic Zn–5Al alloy using 4 M NaOH solution. Owing to the unique lamellar pattern of precursor alloy, selective dissolution of α-Al phase produced Zn lamellar framework with aligned channels, resulting in a highly ordered porous structure. As shown in Figure 1e–g, SEM image of the as-dealloyed pZn reveals a three-dimensional lamellar morphology composed of quasi-periodic channels and Zn ligaments, maintaining the Zn lamellar thickness comparable to that of the precursor alloy.

**Figure 2.**
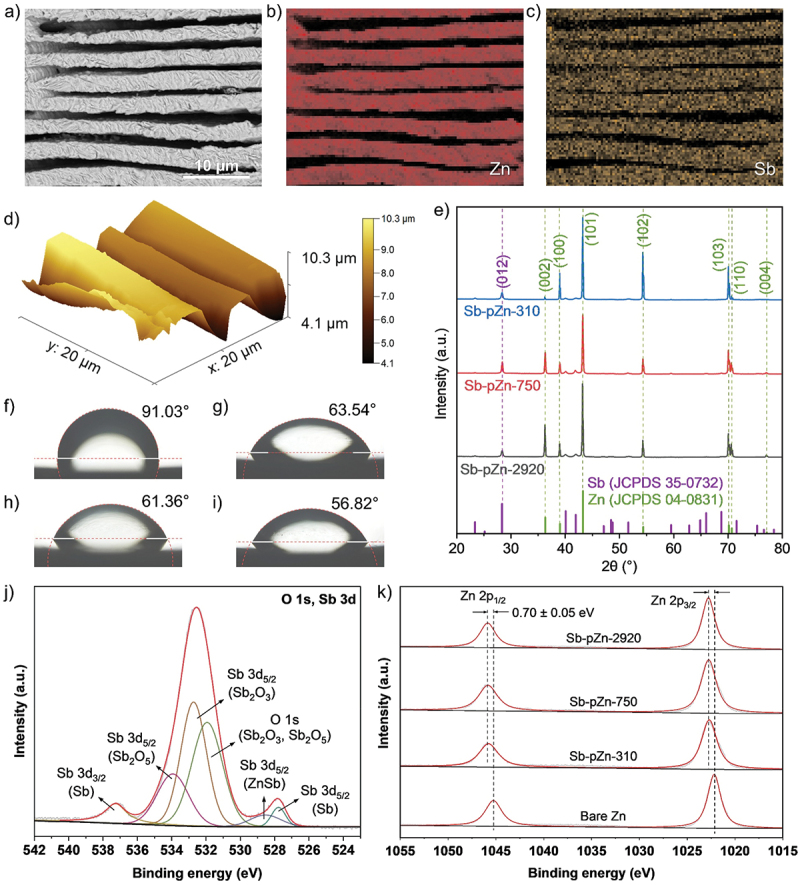
Materials characterization of the modified Zn-based anode. (a) SEM image of Sb-pZn-2920 and the corresponding EDS elemental mappings of (b) Zn and (c) Sb. (d) 3D topographic AFM image of Sb-pZn-2920. (e) XRD patterns of Sb-pZn with reference line patterns of Zn (JCPDS 04–0831) and Sb (JCPDS 35–0732). Contact angle of electrolyte containing Zn-ion on (f) bare Zn, (g) Sb-pZn-310, (h) Sb-pZn-750 and (i) Sb-pZn-2920. (j) high-resolution O 1s and Sb 3d XPS spectra of Sb-pZn-2920. (k) binding energy shift in the Zn 2p region of Sb-pZn.

Then, surface modification of pZn was achieved through a facile chemical displacement reaction. In SbCl_3_-ethanol solution, metallic Sb was uniformly integrated onto pZn sheets by galvanic replacement process. The pZn sheet was immersed in a 0.15-M SbCl_3_–ethanol solution to initiate the reaction, during which Zn was oxidized to Zn^2+^ and Sb^3+^ was reduced and deposited as metallic antimony (3Zn + 2SbCl_3_ → 3ZnCl_2_ +2Sb) [11,39]. The porous structure is fully retained after Sb modification, with Zn lamellar thickness and interlamellar spacing essentially identical to those of pZn (Figure 1h–j). The modification process effectively preserves the characteristic porous structure. SEM micrographs with corresponding EDS mapping in Figure 2a–c and Figure S2 confirm the uniform deposition of Sb, forming a textured layer over the freestanding pZn framework while maintaining the underlying lamellar porosity. AFM imaging (scan size of 20 × 20 μm) further verifies the 3D surface topography, showing that Sb-pZn-2920 (Figure 2d) exhibits higher pit depth (Sv = 3.123 µm) and larger true surface area (591.1 μm^2^) compared with Sb-pZn-750 and Sb-pZn-310 (Figure S3, Table S1). The greater Zn lamellar thickness increases surface roughness, indicating a more developed porous structure and larger surface area. XRD analysis (Figure 2e, Figure S4) confirmed the hybrid structure, displaying two distinct sets of diffraction peaks. The minor peaks at 28.36°, 40.05°, 41.94°, 47.04°, 48.32°, 48.77°, 51.68°, 59.50°, 62.74°, 64.88°, 65.96° and 68.71° can be indexed to (0 1 2), (1 0 4), (1 1 0), (0 1 5), (0 0 6), (1 1 3), (2 0 2), (0 2 4), (1 0 7), (1 1 6), (1 2 2) and (0 1 8) planes of hexagonal Sb, while the remaining major peaks at 36.26°, 38.97°, 43.19°, 54.28°, 70.05°, 70.60° and 77.04° are attributed to (0 0 2), (1 0 0), (1 0 1), (1 0 2), (1 0 3), (1 1 0) and (0 0 4) planes of hexagonal Zn, respectively.

The wettability was evaluated by contact angle measurements with the electrolyte containing Zn ions (8.5 M KOH and 0.6 M ZnO). The bare Zn exhibited a high contact angle of 91.03° (Figure 2f), attributed to its zincophobic planar surface that limits electrolyte interaction. In contrast, the contact angles of pZn-310, pZn-750 and pZn-2920 decreased to 74.42°, 74.10° and 67.43° (Figure S5), respectively, indicating enhanced wettability. Remarkably, after Sb modification, the contact angles further decreased to 63.54° (Sb-pZn-310), 61.36° (Sb-pZn-750) and 56.82° (Sb-pZn-2920), owing to the high surface energy of Sb which promotes electrolyte accessibility (Figure 2g–i). The transition from zincophobicity to zincophilicity is also supported by the uniform porous lamellar architecture that enhances capillary action and liquid-solid interactions [40,41]. The difference in contact angle between solutions containing ZnO and those without was compared for bare Zn, pZn-2920 and Sb-pZn-2920 (Figure S6). As the result, Sb-pZn-2920 shows the most significant decrease from 69.77° (KOH solution) to 56.82° (KOH + ZnO solution), indicating that Sb enhances specific interaction with Zn^2+^-containing electrolyte. The low-resolution XPS survey scan verified the presence of Zn and Sb in their expected regions (Figure S7). Deconvolution of the asymmetric Sb 3d (Figure 2j, Figure S8) and Sb 4d (Figure S9) spectra revealed contributions from both metallic and oxidized species. Metallic Sb was detected that indicates stable integration into the surface, showing that Sb is encapsulated within the uppermost surface layer, while the oxidized Sb species originate from surface oxidation upon exposure to air. Density functional theory (DFT) calculations reported in the literature show that Sb possesses a highly negative Zn adsorption energy (−3.12 eV), indicating strong Zn affinity [18]. Consistent with this prediction, XPS analysis reveals binding energy shifting, suggesting electronic interaction between Zn and Sb, which supports the zincophilic nature of Sb. Figure 2k presents the high-resolution XPS spectra of Zn 2p. A shift toward higher binding energy was observed after Sb modification. Li et al. [42] justified that the difference in Zn and Sb coordination arises from their polarity difference, leading to the formation of polar Zn-Sb bonds. These bonds strengthen adhesion to Zn metal and improve surface stability. The zincophilic nature of Sb promotes interfacial bonding, resulting in the increased Zn binding energy. Furthermore, the shift is also influenced by electronic structure changes induced by Sb incorporation. The presence of Sb alters the electron density distribution around Zn atoms, reducing the outer electron density of Zn and weaken the shielding effect on inner core electrons. Consequently, the core electrons experience stronger attraction from the nucleus, resulting in increased binding energy [43–45]. This phenomenon is consistent with charge transfer effects driven by differences in electronegativity between neighbouring atoms. Electronegativity plays a crucial role in determining core-level binding energies, as it influences charge redistribution. Zn (1.65) is less electronegative than Sb (2.05), leading to partial electron withdrawal from Zn toward Sb [42,46]. This charge transfer reduces electron shielding and increases the effective nucleus attraction on Zn core electrons, further contributing to the observed binding energy shift.

### Electrochemical properties of Sb-pZn

**Figure 3.**
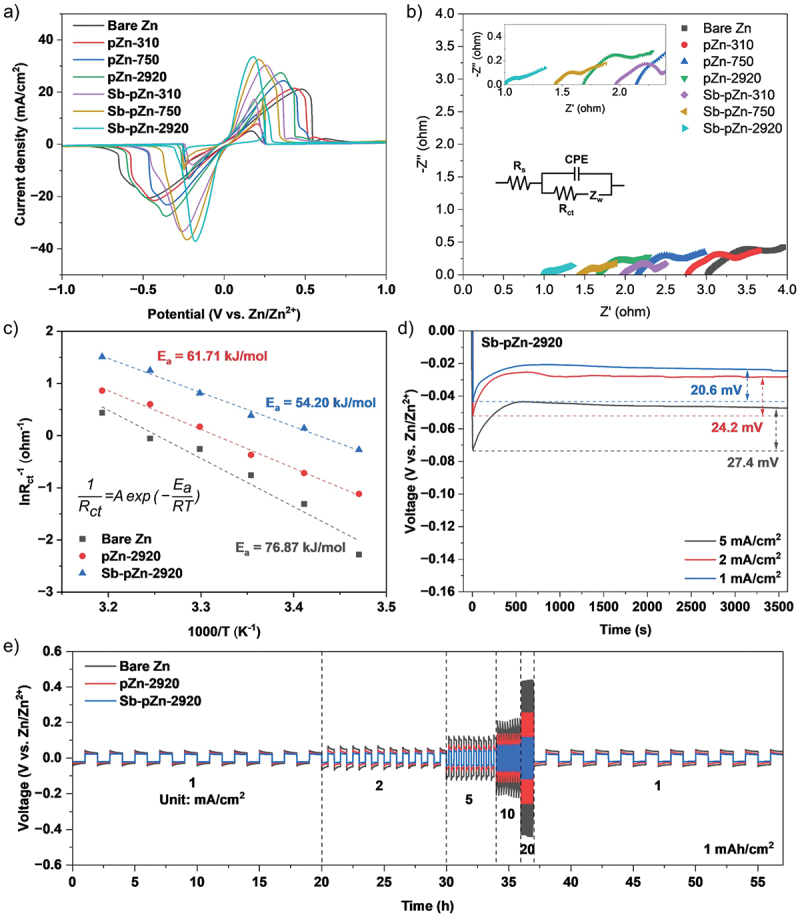
Electrochemical performance of the modified Zn-based anode in symmetric cells. (a) CV curves at scan rate of 10 mV s^−1^. (b) Nyquist plots at temperature of 25°C. (c) Activation energy of zinc deposition on Sb-pZn-2920, pZn-2920 and bare Zn. (d) Nucleation overpotentials of Sb-pZn-2920 at current density from 1 to 5 mA cm^−2^. (e) Voltage profiles of Sb-pZn-2920, pZn-2920 and bare Zn at areal capacity of 1 mAh cm^−2^ and current densities from 1 to 20 mA cm^−2^.

To investigate the Zn stripping-plating behavior, diverse electrochemical measurements were performed on symmetric cells using 8.5 M KOH and 0.6 M ZnO aqueous electrolyte. In the CV curves, Sb-pZn-2920 shows the highest current response and the smallest stripping-plating onset potential separation (Figure 3a, Table S2). A clear correlation is observed, where structures with greater lamellar channels consistently demonstrate superior performance both before and after Sb modification, owing to their more open porous architecture, which offers abundant active surfaces and broad ion diffusion pathways. Given that both modified Sb-pZn and unmodified pZn exhibit nearly identical porous structure, the superior performance of Sb-pZn highlights the critical role of Sb in accelerating Zn deposition kinetics. This improvement is confirmed by EIS analysis (Figure 3b, Figure S10) that shows the introduction of Sb further decreases the R_ct_ by nearly half. The lamellar thickness of 2920 nm demonstrated good performance, showing R_ct_ at 0.64 Ω (pZn-2920) and reduced to the lowest value at 0.29 Ω (Sb-pZn-2920) after Sb modification (Table S3). Similarly, R_s_ decreases with increasing lamellar thickness, reaching as low as 1.02 Ω (Sb-pZn-2920) after Sb modification. The larger Zn lamellar network provides better electronic conductivity, while the strong zincophilicity of Sb further promotes interfacial reaction kinetics. To gain deeper understanding of the accelerated charge-transfer process, impedance measurements were carried out at varied temperatures. Sb-pZn-2920 exhibits consistently lower R_ct_ across all temperature ranges (Figure S11, Table S4). The activation energy (E_a_) was subsequently determined using the Arrhenius equation, where it represents the desolvation energy barrier that Zn^2+^ ions must overcome during migration. Based on the temperature-dependent Nyquist plots, E_a_ was calculated using the Arrhenius equation (1/*R_ct_* = *A*exp(−*E_a_*/*RT*)), where *T*, *R*, *A* and *R_ct_* correspond to the absolute temperature, the gas constant, the frequency factor and charge transfer resistance, respectively [10,47,48]. From the linear fitting of ln(1/*R_ct_*) versus 1000/*T* (Figure 3c), the *E_a_* of Sb-pZn-2920 was determined to be 54.20 kJ mol^−1^, which is lower than pZn-2920 (61.71 kJ mol^−1^) and bare Zn (76.87 kJ mol^−1^). These results indicate that Sb-pZn-2920 facilitates Zn^2+^ desolvation and accelerates ion migration at the electrolyte–electrode interface. The strong zincophilicity of Sb lowers the *E_a_* by weakening the coulombic interaction between Zn^2+^ and its solvation sheath.

Nucleation overpotential further evaluates the zincophilicity of the modified anode. The reduced nucleation overpotential corresponds to a lower nucleation energy barrier [49–51]. The pZn-2920 shows lower nucleation overpotential than bare Zn, benefiting from the porous lamellar framework (Figure S12), while Sb-pZn-2920 shows further lower nucleation overpotential than pZn-2920, confirming contributions of Sb in Zn deposition (Figure 3d). The voltage profiles of symmetric Sb-pZn-2920, pZn-2920 and bare Zn cells at varying current densities of 1–20 mA cm^−2^ are shown in Figure 3e. Sb-pZn-2920 displays the flattest voltage plateaus with a small overpotential of 25.5 mV at 1 mA cm^−2^, which is 7.8 and 17.7 mV lower than those of pZn-2920 (33.3 mV) and bare Zn (43.2 mV), respectively. This reflects the synergistic effects of strong zincophilicity of Sb and the extensive electroactive surface area of the lamellar structure, enabling Zn stripping and plating with minimal polarization. Consistent with CV analysis, even as the current density increases to 2, 5, 10 and 20 mA cm^−2^, Sb-pZn-2920 maintains small overpotentials of 30.3, 47.6, 76.3 and 144.4 mV, respectively, which remain significantly lower than pZn-2920 and bare Zn. Exchange current density reflects the electrochemical reaction rate at equilibrium potential and reveals the Zn plating-stripping kinetics. It is determined using the equation *i* = 2ioF*η*/(RT), where *i* denotes the current density, *i_o_* is the exchange current density and *η* represents the total overpotential [10,52]. Figure S13 shows the linear fitted plots of overpotential versus current density. The exchange current densities of Sb-pZn-2920, pZn-2920 and bare Zn are determined to be 2.302, 1.322 and 0.672 mA cm^−2^, respectively. The higher exchange current density of Sb-pZn-2920 indicates reduced electrochemical polarization, improved electron transfer and accelerated Zn plating-stripping kinetics.

**Figure 4.**
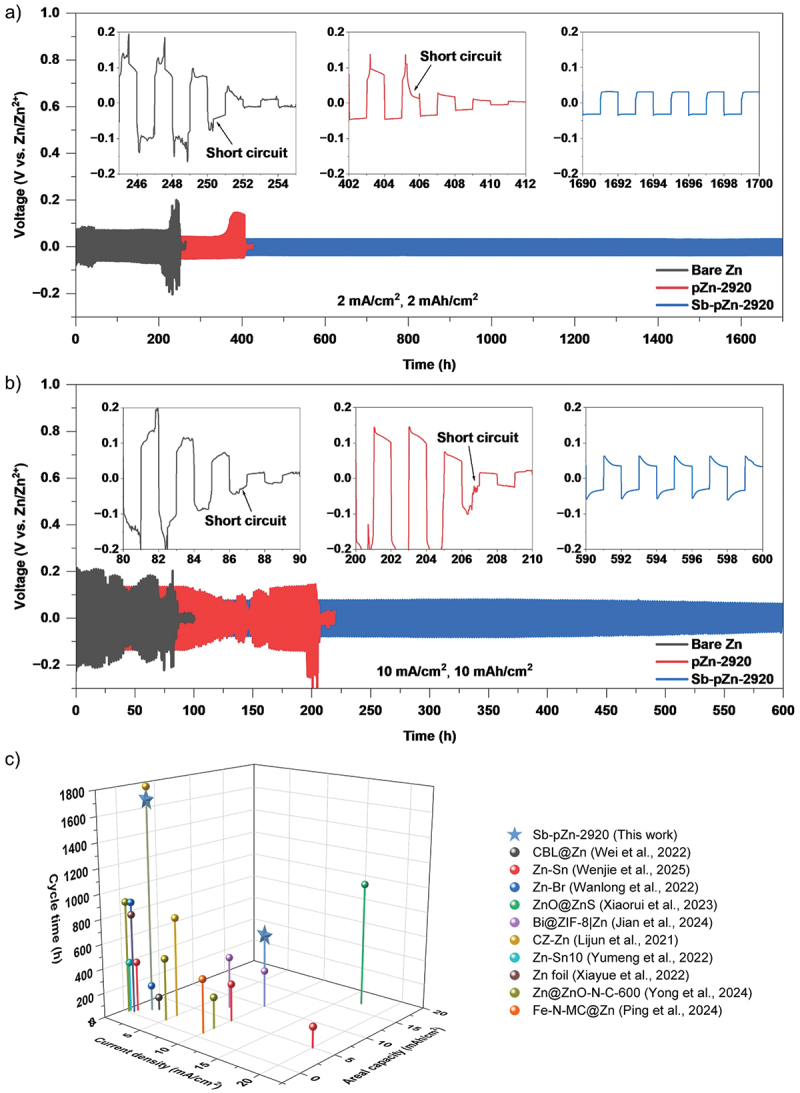
Long-term cycling performance of Sb-pZn-2920, pZn-2920 and bare Zn symmetric cells at (a) 2 mA cm^−2^ and 2 mAh cm^−2^, (b) 10 mA cm^−2^ and 10 mAh cm^−2^. (c) Comparison of cycling performance between Sb-pZn-2920 and other advanced Zn anodes in literatures.

To study the effect of the modified anode on plating-stripping stability, galvanostatic charge–discharge cycle tests were performed. At a current density of 2 mA cm^−2^ and areal capacity of 2 mAh cm^−2^, Sb-pZn-2920 shows the most stable cycling, with low overpotential of 31.7 mV and prolonged lifespan of up to 1700 h (Figure 4a). In contrast, pZn-2920 and bare Zn experience serious voltage fluctuations, with sudden voltage drops occur after 405 h and 250 h, respectively, indicating short circuit caused by rampant Zn dendrite growth. Additionally, at high current density of 10 mA cm^−2^ and areal capacity of 10 mAh cm^−2^, Sb-pZn-2920 continues to maintain stable cycling for up to 600 h with overpotential of 64.2 mV, which remains lower than the initial overpotential of pZn-2920 and bare Zn (Figure 4b). Under the same conditions, pZn-2920 and bare Zn show severe voltage fluctuations, with rapid short circuit occur after only 206 h and 86 h, respectively. Similarly, the failure mechanisms can be attributed to the accumulation of Zn dendrites, which eventually penetrate the separator and cause cell degradation [53,54]. Table S5 benchmarks the cycling performance of Sb-pZn-2920 anode against state-of-the-art Zn anodes reported for symmetric cells across diverse Zn-based energy storage systems. Given the similar electrochemical behavior of Zn anodes in alkaline electrolytes, this comparison provides a meaningful view of their overall performance. Compared to other modification strategies, Sb-pZn-2920 outperforms most reported anodes by up to twofold (Figure 4c). The exceptional cycling stability highlights both the superior reversibility and structural robustness of Sb-pZn-2920, establishing it as one of the most promising anode candidates for next-generation aqueous ZAB.

**Figure 5.**
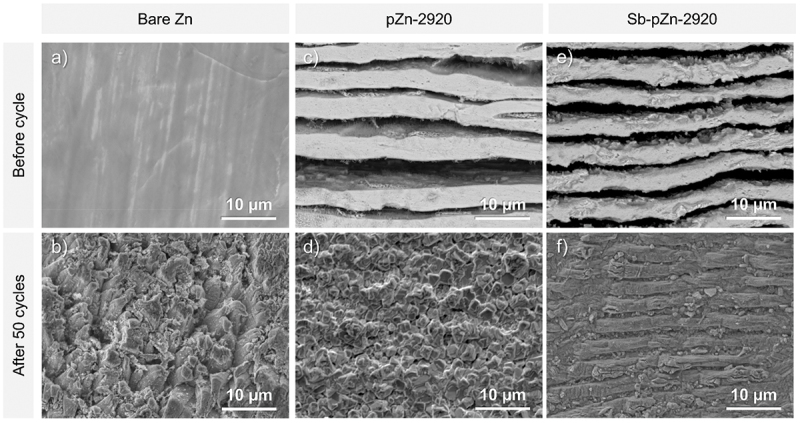
SEM images of the surface of (a–b) bare Zn, (c–d) pZn-2920 and (e–f) Sb-pZn-2920 before and after 50 cycles at current density of 2 mA cm^−2^ and areal capacity of 2 mAh cm^−2^.

The exceptional long-term cycling performance of Sb-pZn-2920 at high current density demonstrates that the modified anode effectively suppresses Zn dendrite growth and significantly enhances Zn^2+^ plating-stripping behavior. After 50 h of stripping-plating cycles at current density of 10 mA cm^−2^ and areal capacity of 10 mAh cm^−2^, surface morphology of the electrode was examined. Bare Zn develops uneven protrusions that readily initiate dendritic growth (Figure 5a–b). In comparison, pZn-2920 shows a rough surface with regular micron-sized bumps, indicating that the lamellar pores help suppress dendrite formation by providing confined spaces for controlled Zn deposition during the initial cycles (Figure 5c–d). Under the same conditions, Zn deposition on Sb-pZn-2920 appears smooth and dense without large protrusions, confirming that Sb-pZn-2920 promotes uniform Zn growth (Figure 5e–f). The incorporation of Sb within the lamellar porous structure homogenizes the electric field distribution and Zn^2+^ flux. The porous framework lowers the local current density due to enlarged surface area, while zincophilic Sb sites facilitate Zn^2+^ adsorption, leading to uniform Zn deposition [18,45].

**Figure 6.**
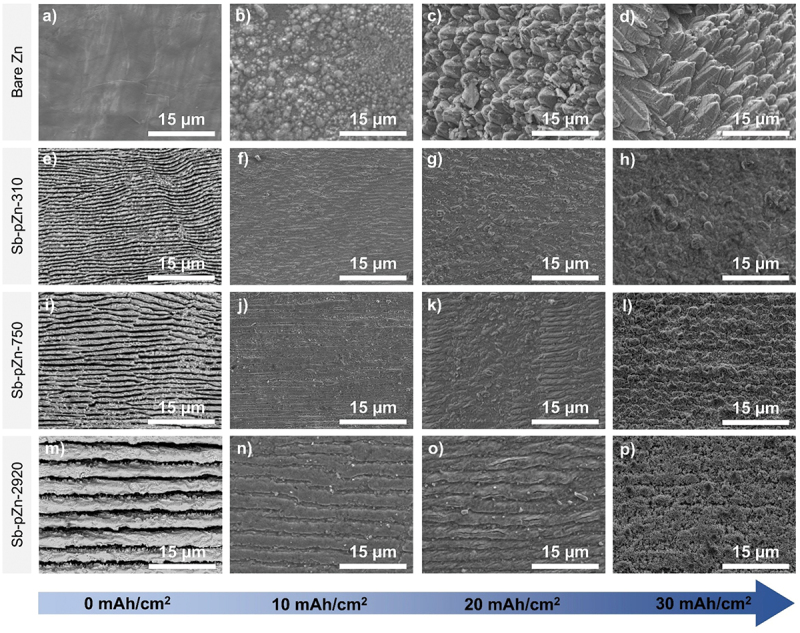
SEM images of the surface of (a–d) bare Zn, (e–h) Sb-pZn-310, (i–l) Sb-pZn-750 and (m–p) Sb-pZn-2920 before and after Zn deposition at areal capacities from 0 to 30 mAh cm^−2^.

To further study the dendritic growth, ex-situ SEM was conducted to observe the morphological evolution of Zn electrodeposition at areal capacities ranging from 0 to 30 mAh cm^−2^. Before deposition, the bare Zn surface appeared smooth (Figure 6a). However, in the initial plating at 10 mAh cm^−2^, distinct particle-like dendrites began to form, resulting in a rough surface with localized regions of intense dendritic growth (Figure 6b). As the areal capacity increased, these dendritic structures expanded progressively, driven by localized electric field. At 20 mAh cm^−2^, numerous protuberances were observed (Figure 6c), which evolved into well-defined dendritic formations extending outward from the bulk Zn surface. Branching structures including herringbone-type needle formations were clearly observed. When the areal capacity reached 30 mAh cm^−2^, the dendritic formations developed into a dense, forest-like network that nearly covered the entire Zn surface (Figure 6d). Rather than merging into a uniform layer, the dendrites remained distinct and continued to grow independently. The sharp dendrite tips exhibited the tip effect where Zn atomic clusters preferentially accumulated and reinforce the self-perpetuating growth of dendrites [55,56]. At this stage, the deposition process became highly uncontrolled with extensive Zn protrusions developing 3D tree-like dendritic morphology. The formation of these dendrites was primarily attributed to the limited nucleation sites and poor surface wettability which led to the localized accumulation of Zn^2+^ ions and uneven Zn deposition [57–59]. In contrast, Zn electrodeposited on Sb-pZn-310, Sb-pZn-750 and Sb-pZn-2920 appeared smooth across all plating capacities (Figure 6e–p). At the initial plating of 10 mAh cm^−2^, a dense film-like Zn layer forms, effectively suppressing dendrite growth. As the plating capacity increases to 20 mAh cm^−2^, the Zn layer remains uniform with minimal surface irregularities. Even at a high areal capacity of 30 mAh cm^−2^, the surface stays compact and free of visible dendritic features. Overall, the modified anode demonstrates excellent uniformity, ensuring the formation of stable, dendrite-free Zn layers under high Zn loading.

### Electrochemical performance of ZAB full cells

ZAB plays a critical role in the proposed self-powered energy system. To evaluate the practical feasibility of the modified anode, ZAB full cells were assembled (Figure S14) with MnO_2_ and Ni(OH)_2_ as bifunctional electrocatalysts for the oxygen reduction (ORR) and oxygen evolution (OER) reactions, respectively [60,61], supported on the PTFE-treated Toray carbon paper as the air cathode and paired with the as-prepared anode in the aqueous electrolyte containing 8.5 M KOH and 0.6 M ZnO. The surface morphology and crystalline structure of the air cathode were characterized by SEM-EDS and XRD as shown in Figure S15 and Figure S16, respectively.

**Figure 7.**
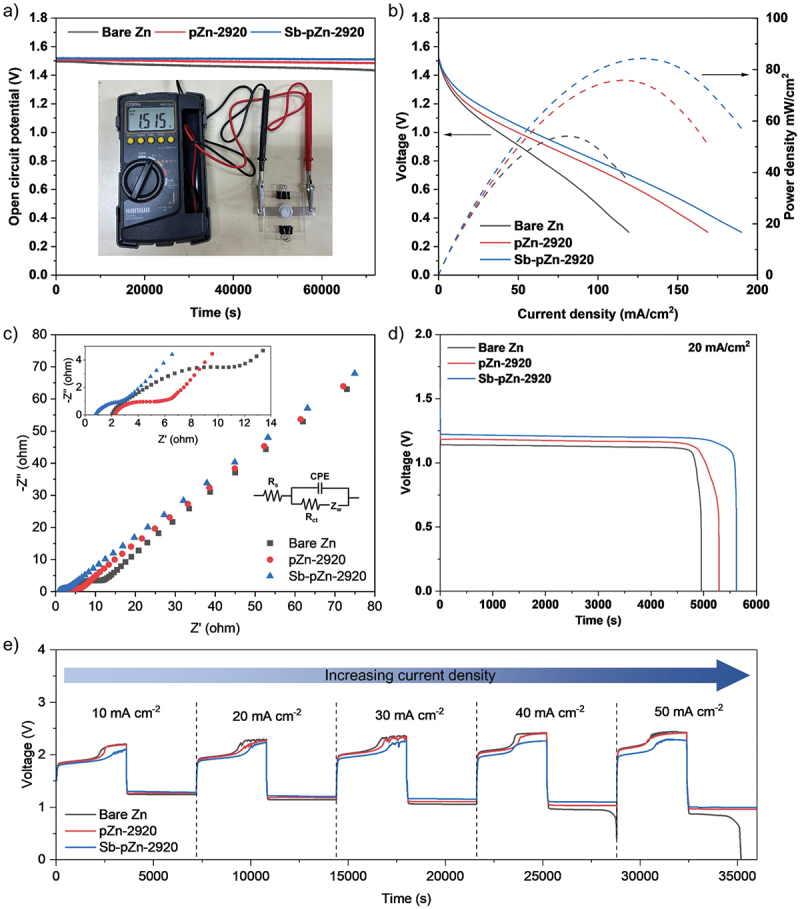
Electrochemical performance of Sb-pZn-2920||O_2_, pZn-2920||O_2_ and bare Zn||O_2_ full cells. (a) Open circuit potential. (b) Discharge polarization profiles with the corresponding power densities. (c) Nyquist plots. (d) Galvanostatic discharge profiles at current density of 20 mA cm^−2^. (e) Charge–discharge profiles of Sb-pZn-2920||O_2_ at current densities from 10 to 50 mA cm^−2^.

Sb-pZn-2920||O_2_ cell delivered an OCV of 1.51 V (Figure 7a), consistent with values reported for typical ZABs. When exposed to ambient air for 20 h, Sb-pZn-2920||O_2_ cell maintains stable voltage, indicating excellent electrode stability. In contrast, the OCV of pZn-2920||O_2_ and bare Zn||O_2_ cells slightly decrease from 1.50 to 1.43 V and 1.51 to 1.48 V respectively, primarily due to self-discharge resulting from side reactions, surface corrosion and passivation on the planar Zn electrode. The presence of Sb effectively prevents localized self-passivation by reducing the tendency of Zn to form ZnO and slowing down surface oxidation [62]. As the result, the surface has low passivity, allowing Sb-pZn-2920 to maintain superior stability in strong alkaline electrolyte compared with pZn-2920 and bare Zn. To verify the suppression of side reactions, Tafel polarization measurements were performed in three-electrode system using graphite as the counter electrode and Ag/AgCl as the reference electrode. Sb-pZn-2920 shows corrosion current density (i_corr_) of 0.02291 A cm^−2^ (Figure S17), lower than pZn-2920 (0.02911 A cm^−2^) and bare Zn (0.03476 A cm^−2^). Meanwhile, the corrosion potential (E_corr_) was slightly more positive (−1.410 V) compared with pZn-2920 (−1.418 V) and bare Zn (−1.450 V), indicating improved thermodynamic stability. The lower i_corr_ and more positive E_corr_ confirm that Sb-pZn-2920 possesses superior corrosion resistance. The Sb-enriched surface reduces direct contact between the active Zn and electrolyte, effectively mitigating corrosion [62,63]. In addition, the presence of Sb may modify the equilibrium potential through surface alloying effects, which contributes to the observed shift in E_corr_ and further suppresses side reactions such as hydrogen evolution. Discharge polarization curves further demonstrate the superior electrochemical performance of the modified anode. Bare Zn||O_2_ cell experienced significant voltage drop with increasing current density (Figure 7b), indicating limited anode utilization. In comparison, Sb-pZn-2920||O_2_ cell showed minimal potential decay, maintaining discharge potential of 0.3 V even at high current density of 190 mA cm^−2^. The corresponding power density shows high peak output of 84.36 mW cm^−2^, sufficient to power compact electronic devices such as digital clocks, LED flashlights, portable fans and sensors. From Figure S18, the overlapping CV profiles with stable redox peak positions over the first four cycles indicate highly reversible and stable electrochemical reactions. EIS analysis shows that the R_s_ and R_ct_ of Sb-pZn-2920||O_2_ cell are 0.75 Ω and 3.57 Ω respectively, lower than pZn-2920||O_2_ and bare Zn||O_2_ (Figure 7c), indicating improved charge-transfer kinetics and reduced interfacial impedance. As shown in Figure 7d, the galvanostatic discharge profiles show stable voltage plateau for over 1 h at current density of 20 mA cm^−2^, indicating efficient anode utilization and sustained ORR activity at the air cathode. Sb-pZn-2920||O_2_ cell maintains steady potential at 1.21 V, higher than pZn-2920||O_2_ at 1.17 V and bare Zn||O_2_ at 1.13 V, consistent with the results of polarization test and CV. As the discharge progresses, Zn is gradually oxidized to form soluble zincate ions in the alkaline electrolyte. When the concentration of zincate ions exceeds the solubility limit, ZnO precipitates on the anode surface, blocking active sites and hindering further Zn dissolution. The accumulation of these insulating products, along with continuous Zn consumption, results in gradual voltage drop [64,65]. Once all active Zn is depleted, the sharp voltage drop to 0 V occurs, indicating cell termination and completion of the discharge process. Specific capacity was determined based on the discharge time and normalized to the mass of zinc consumed, providing measure of the active material utilization. As summarized in Table S6, Sb-pZn-2920||O_2_ cell achieves specific capacity of 580.7 mAh g^−1^, higher than bare Zn||O_2_ at 512.0 mAh g^−1^ and pZn-2920||O_2_ at 546.7 mAh g^−1^. Additionally, the anode utilization is around ~14%, which falls within the normal range and generally considered acceptable for conventional static ZABs under laboratory conditions. This result is consistent with previous report [66], indicating that the present system shows comparable anode efficiency to state-of-the-art ZABs. The enhanced Zn stripping-plating behaviour of the modified anode enables Sb-pZn-2920||O_2_ cell to operate stably across varied charge–discharge current densities from 10 to 50 mA cm^−2^. Notably, it always shows smaller potential gap than both pZn-2920||O_2_ and bare Zn||O_2_ cells (Figure 7e), especially at high current densities, confirming its superior reaction kinetics.

**Figure 8.**
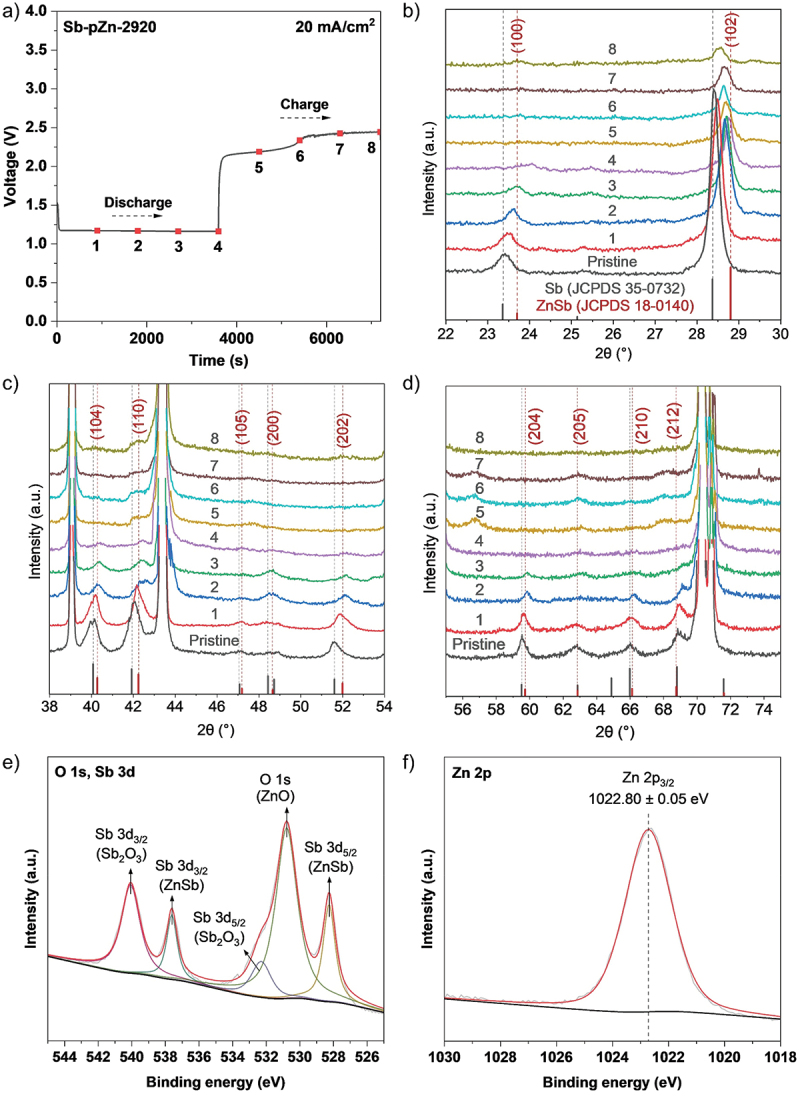
Schematic analysis of structural and composition changes of Sb-pZn-2920 during cycling. (a) Charge–discharge curves at 20 mA cm^−2^. XRD patterns of Sb-pZn-2920 at various charge–discharge states within (b) 22–30°, (c) 38–54° and (d) 55–75°. High-resolution (e) O 1s, Sb 3d and (f) Zn 2p XPS spectra of Sb-pZn-2920 after cycling.

**Figure 9.**
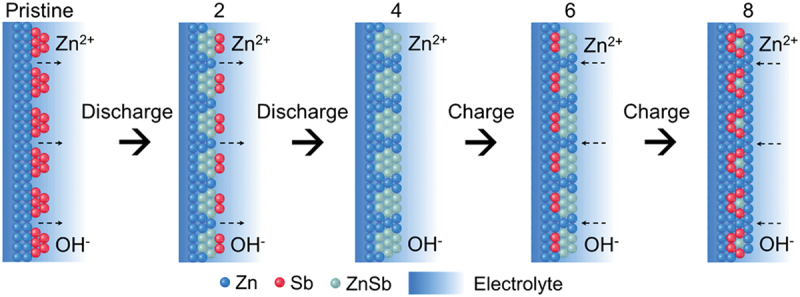
Schematic illustration of the structural and compositional changes of Sb-pZn-2920 during cycling based on the XRD results.

To study deeper insight into the Zn storage mechanism, ex-situ XRD was conducted to examine the structural and composition changes of the modified anode during cycling. Sb-pZn-2920||O_2_ cell was cycled through eight set discharge–charge points (Figure 8a), after which the anodes were rinsed with deionized water and ethanol and dried at 40°C under vacuum. The resulting XRD patterns at the eight endpoints within the initial cycle are presented in Figure 8b–d. The pristine Sb-pZn-2920 anode shows combined peaks of pure crystalline Zn and Sb. After discharge for 1 h, the high-intensity peak of Sb located at 28.38° shifts positively to 28.76°, indicating the insertion of Zn atoms into the Sb lattice and the formation of new alloy phase (Figure 8b) [39,63]. The characteristic peak of ZnSb (JCPDS 18-0140) is detected. Indeed, according to the Zn–Sb binary-phase diagram (Figure S19), the newly formed phase is indexed to Zn–Sb intermetallic compound, which can form at ambient temperature and remain stable at relatively low temperatures. In Figure 8c–d, the characteristic peaks at 23.74, 40.30°, 42.34°, 47.18, 48.65, 52.16, 59.87°, 62.84°, 66.26° and 69.10° also correspond to ZnSb, matching well with the standard PDF card of ZnSb (JCPDS 18–0140). These results confirm that Sb is alloyed with Zn to form ZnSb phase during the discharge process. The peak intensity of Sb gradually decreased, indicating that Sb was actively consumed [39]. During the subsequent charging process, the characteristic ZnSb peak slightly shifts from 28.76° to 28.54°, demonstrating the reversible alloying reaction between Sb and Zn. However, the peak location does not revert to pristine Sb-pZn-2920 (28.38°) due to crystalline lattice change after Sb alloying. According to Tian et al. [39], Sb exhibits good reversibility in alloying reactions. The charge–discharge mechanism of alloying-type antimony electrodes for Zn-ion batteries can be summarized as Sb + xZn ↔ Zn_x_Sb. Over extended operation, Sb continues to participate in reversible alloying to form ZnSb until it is gradually depleted. Even so, the chemical stability remains high, as confirmed by the long-term GCD test in symmetric cell. Regulating the first cycle is particularly meaningful, as it directly influences subsequent cycling behavior. This structural change contributes to the formation of stable zincophilic interface, which enhances reversibility and overall cycling durability of the anode [67,68]. The surface alloying effect between Sb and Zn modifies the local equilibrium potential, suppressing side reactions such as corrosion and hydrogen evolution and improving the electrochemical stability. XPS analysis further supports the phase evolution after cycling process. High-resolution Sb 3d spectrum (Figure 8e) shows strong ZnSb signal, consistent with the ex-situ XRD results. Peaks at 537.6 ± 0.05 eV (Sb 3d3/2) and 528.2 ± 0.05 eV (Sb 3d5/2) belongs to the metallic Sb species from the ZnSb intermetallic compound. Additionally, the Zn 2p3/2 peak remains at a high binding energy of 1022.8 ± 0.05 eV (Figure 8f), comparable to that of the pristine Sb-pZn-2920, confirming the formation of stable zincophilic interface. The schematic illustration of the structural and compositional changes of Sb-pZn-2920 during cycling is shown in Figure 9.

**Figure 10.**
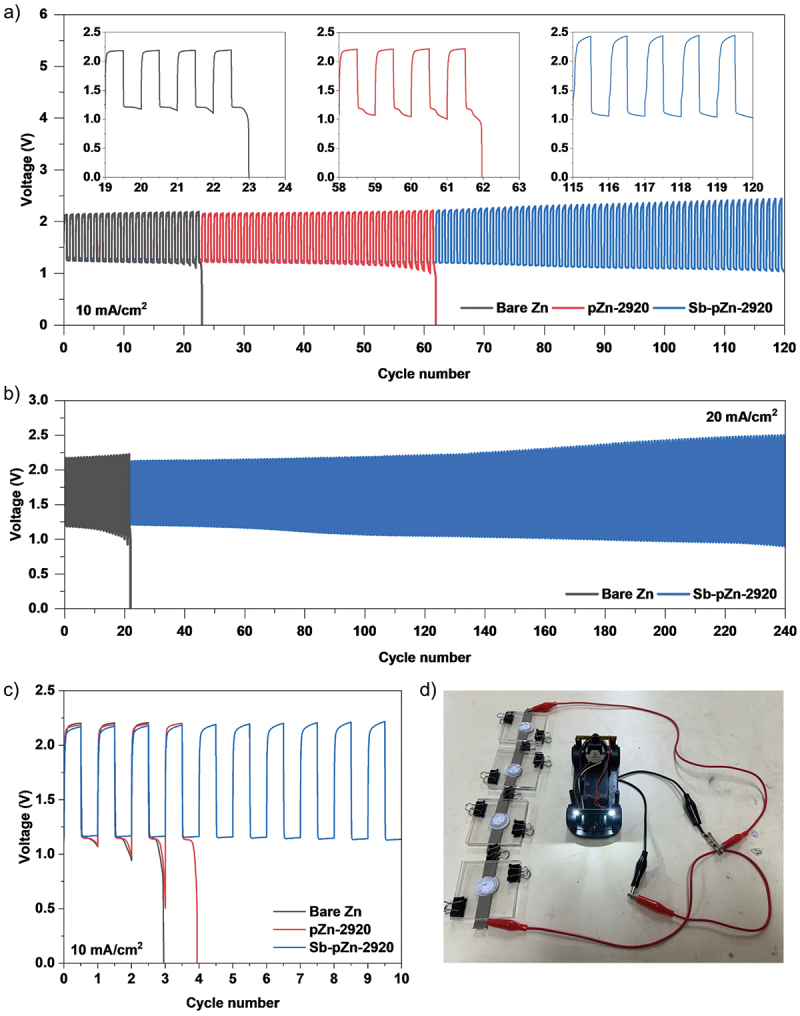
Long-term cycling performance of Sb-pZn-2920||O_2_, pZn-2920||O_2_ and bare Zn||O_2_ full cells at current density of (a) 10 mA cm^−2^ and (b) 20 mA cm^−2^ with 1 h per cycle. (c) Cycling performance of reassembled Sb-pZn-2920||O_2_, pZn-2920||O_2_ and bare Zn||O_2_ full cells at 10 mA cm^−2^. (d) Photograph of a toy car powered by four series-connected Sb-pZn-2920||O_2_ batteries.

To verify the cycling stability, galvanostatic charge–discharge tests were conducted, with each cycle consist of 30-min charge followed by 30-min discharge at a current density of 10 mA cm^−2^ under ambient air conditions. Sb-pZn-2920||O_2_ cell nearly shows stable charge and discharge potentials at 2.44 V and 1.04 V, respectively, for up to 120 continuous cycles (Figure 10a). In contrast, pZn-2920||O_2_ and bare Zn||O_2_ cells failed after 62 h and 23 h, respectively, as indicated by a sharp voltage drop to 0 V due to anode degradation. Even at an ultrahigh current density of 20 mA cm^− 2^, Sb-pZn-2920||O_2_ cell shows remarkable long-term cycling stability, operating steadily for over 240 h (Figure 10b), more than 10 times longer than the bare Zn||O_2_ cell, which suffers rapid failure after only 22 h under the same conditions. Table S7 benchmarks the cycling performance against previously reported alkaline ZAB systems. Compared to other modification strategies, Sb-pZn-2920||O_2_ cell outperforms most reported ZABs in charge–discharge cycling at high current density. This outstanding performance underscores its promise for rapid energy delivery and fast-charging applications, addressing the critical need in modern energy storage technologies [69,70]. To further identify the main factor contributing to cell failure, post-mortem analysis was conducted. The failed pZn-2920||O_2_ and bare Zn||O_2_ cells were disassembled (Figure S20), and the components including the anode and air cathode were separated for further examination. Subsequently, new cells were assembled using the used anodes in combination with new separators, fresh electrolytes and new air cathodes [71]. As shown in Figure 10c, both reconstructed pZn-2920||O_2_ and bare Zn||O_2_ cells operated for only four and three cycles, respectively, before failing, confirming that degradation of the anode is the major cause of performance decay during repeated charge–discharge cycling. The practical applicability of the modified anode was further demonstrated through a simple prototype test. As illustrated in Figure 10d, four ZAB cells connected in series using Sb-pZn-2920 anode successfully powered a toy car, which operated steadily and continuously for over 1 h in ambient air.

## Conclusion

In conclusion, the self-supported Sb-pZn anode has been successfully developed as a reversible, dendrite-free and high-performance hostless Zn anode for aqueous alkaline rechargeable ZAB. The well-engineered structures with large interconnected lamellar network exhibit superior performance owing to their more open porous architecture, which provides abundant active sites and broad ion diffusion pathways. Since both Sb-pZn and unmodified pZn possess nearly identical porous structures, the enhanced performance of Sb-pZn underscores the critical role of Sb in accelerating Zn deposition kinetics through its strong zincophilicity and improved interfacial reaction dynamics. The Sb-pZn-2920 anode delivers low overpotential of 31.7 mV for up to 1700 h at 2 mA cm^−2^ in symmetric cells. When assembled into full ZAB cell, the Sb-pZn-2920||O_2_ achieves high specific capacity of 580.7 mAh g^−1^, a high power density of 84.36 mW cm^−2^ and charge–discharge cycle life 10 times longer than that of the bare Zn||O_2_ cell. The synergistic effects of porous lamellar framework and Sb modification present a practical strategy for overcoming the challenges of planar Zn anodes, offering a versatile route for tailoring the surface chemistry and microstructure of metal-based electrodes. These findings represent a significant step toward realizing safe, durable and efficient aqueous Zn energy storage systems for large-scale applications.

## Supplementary Material

Supplemental Material
