# Supplementary material for: Antimony-modified porous lamellar zinc as reversible and stable anode for high-performance alkaline aqueous zinc–air battery
Source: Sci Technol Adv Mater. 2026 May 7;27(1):2658327. doi: 10.1080/14686996.2026.2658327 (PMC13159586; doi:10.1080/14686996.2026.2658327)
Supplement: Supplemental Material [file TSTA_A_2658327_SM5778.pdf]

Supporting Information

Antimony-modified porous lamellar zinc as reversible and stable anode for high-performance alkaline aqueous zinc-air battery

Muhammad Afiq Irfan Mohd Shumiri[a], Hikari Sakaebe[b], Abdillah Sani Mohd Najib[a,c], Nor Akmal Fadil[a,c,*]

[a]*Materials Research and Consultancy Group, Faculty of Mechanical Engineering, Universiti Teknologi Malaysia, 81310, Johor Bahru, Malaysia;* [b]*Institute for Materials Chemistry and Engineering, Kyushu University, Kasuga-koen 6-1, Kasuga, Fukuoka 816-8580, Japan;* [c]*Department of Materials, Manufacturing and Industry, Faculty of Mechanical Engineering, Universiti Teknologi Malaysia, 81310 Johor Bahru, Malaysia*

*corresponding author e-mail: norakmal@utm.my

Table S1. AFM true surface area, maximum pit depth and RMS roughness

Sample	***Scan size (µm)***	***Projected surface area (µm^2)***	***True surface area (µm^2)***	***Max. pit depth, S_v (µm)***	***RMS roughness, S_q (µm)***
Sb-pZn-310	20 × 20	400	430.0	0.457	0.060
Sb-pZn-750	20 × 20	400	434.9	0.760	0.098
Sb-pZn-2920	20 × 20	400	591.1	3.123	1.156

Table S2. Electrochemical parameters from CV measurements in symmetric cells.

Working electrode	*Anodic peak*		*Cathodic peak*	
	Potential (V)	*Current density (mA cm⁻²)*	*Potential (V)*	*Current density (mA cm⁻²)*
Bare Zn	0.48	21.11	-0.47	-20.60
pZn-310	0.43	21.48	-0.43	-21.56
pZn-750	0.36	24.34	-0.35	-23.15
pZn-2920	0.35	27.39	-0.36	-27.53
Sb-pZn310	0.26	30.22	-0.26	-33.33
Sb-pZn-750	0.21	32.48	-0.23	-36.53
Sb-pZn-2920	0.18	33.47	-0.18	-37.18

Table S3. Electrochemical parameters from EIS measurements in symmetric cells.

Working electrode	***R_s (Ω)***	***R_{ct} (Ω)***
Bare Zn	3.03	1.22
pZn-310	2.78	0.88
pZn-750	2.15	0.79
pZn-2920	1.70	0.64
Sb-pZn310	2.00	0.45
Sb-pZn-750	1.45	0.33
Sb-pZn-2920	1.02	0.29

Table S4. Electrochemical parameters from EIS measurements at different temperatures.

Temperature (°C)	***Bare Zn***		***pZn-2920***		***Sb-pZn-2920***	
	R_s (Ω)	R_{ct} (Ω)	R_s (Ω)	R_{ct} (Ω)	R_s (Ω)	R_{ct} (Ω)
15	6.558	9.76	4.257	3.052	2.109	1.312
20	4.399	3.702	3.596	2.05	1.726	0.866
25	3.047	2.137	2.521	1.447	1.501	0.681
30	2.199	1.29	1.794	0.842	0.862	0.443
35	1.554	1.056	1.272	0.548	0.598	0.287
40	0.927	0.645	0.871	0.422	0.495	0.221

Table S5. Cycling performance comparison of advanced Zn anodes reported in recent literature.

Modification strategy	***Battery system***	***Anode***	***Electrolyte***	***Current density (mA cm^{-2})***	***Areal capacity (mAh cm^{-2})***	***Half-cell lifespan (h)***	***Ref.***
Artificial interphase layer	ZAB	CBL@Zn	KOH + $Zn(AC)_2$	2.5	2.5	100	[1]
Alloying anode	Zn/NCP @PQx	Zn-Sn	KOH + $Zn(AC)_2$	1	1	400	[2]
				10	5	300	
				20	5	165	
Electrolyte engineering	Zn/MnO_2	Zn–Br	KOH+ KBr	1	0.5	900	[3]
				2	2	200	
Zincophilic surface	Zn/Ni	ZnO@ZnS	KOH + ZnO	17	17	1000	[4]
Porous structure	Zn/Ni	Bi@ZIF-8|Zn	KOH + ZnO	7.5	7.5	420	[5]
				10	10	300	
Alloying anode	Ni3S2@ PANI//C Z-Zn	CZ-Zn	KOH + $Zn(AC)_2$	5	2.5	800	[2]
				2	2	1800	
Alloying anode	ZAB	Zn-Sn10	KOH	0.5	0.5	400	[6]
Electrolyte engineering	ZAB	Zn foil	PVA–KOH	1	0.5	800	[7]
Artificial interphase layer	ZAB	ZnO-N-C-600	KOH	0.5	0.25	900	[8]
				5	1	500	
				10	2.5	250	
Artificial interphase layer	ZAB	Fe-N-MC@Zn	KOH	10	1	430	[9]
Porous zincophilic surface	ZAB	Sb-pZn-2920	KOH + ZnO	10	10	600	*This work*
				2	2	1700	

Table S6. Specific capacity and anode utilization of Sb-pZn-2920||O2, pZn-2920||O2 and bare Zn||O2 cell at 20 mA cm^{-2} discharge current density.

ZAB cell	*Current density (mA cm^{-2})*	*Dis. time (s)*	*Weight of anode (g)*		*Specific capacity (mAh g^{-1})*	*Anode utilization (%)*
			Before dis.	*After dis.*		
Bare Zn||O_2	20	4954	0.721	0.626	512.0	13.18
pZn-2920||O_2	20	5290	0.603	0.508	546.7	15.75
Sb-pZn-2920||O_2	20	5619	0.676	0.581	580.7	14.05

Table S7. Cycling performance comparison of advanced Zn anodes in alkaline ZAB system.

Modification strategy	***Battery system***	***Anode***	***Electrolyte***	***Current density (mA cm^{-2})***	***Full cell lifespan***	***Ref.***
Porous structure	ZAB	NP Zn	KOH + $Zn(AC)_2$	10	80 h	[10]
Porous structure	ZAB	Porous Zn0.01	KOH	5	65 cycles	[11]
Porous structure	ZAB	3DP-ZAB	KOH + $Zn(AC)_2$	5	370 cycles	[12]
Alloying anode	ZAB	Ag-modified Zn	KOH + $Zn(AC)_2$	25	140 h	[13]
Porous structure	ZAB	MXene/Zn	KOH + PVA	3	50 h	[14]
Artificial interphase layer	ZAB	CBL@Zn	KOH + $Zn(AC)_2$	1	450 h	[1]
Surface coating	ZAB	TAM-Tp-Zn	KOH + $Zn(AC)_2$	2	600 h	[15]
Porous structure	ZAB	Zn nanoflakes	KOH + $Zn(AC)_2$	5	80 h	[16]
Alloying anode	ZAB	$Zn_{1.5}Bi$ powder	KOH + KF + K_2CO_3	25	300 cycles	[17]
Surface coating	ZAB	ZnO-N-C-600	KOH	5	300 h	[8]
Artificial interphase layer	ZAB	CBL@Zn	KOH + $Zn(AC)_2$	1	500 h	[1]
Alloying anode	ZAB	Zn-Sn10	KOH	5	200 h	[6]
Artificial interphase layer	ZAB	Fe-N-MC@Zn	KOH	5	500 h	[9]
Porous zincophilic surface	ZAB	Sb-pZn-2920	KOH + ZnO	10	120 cycles / 120 h	*This work*
				20	240 cycles / 240 h	

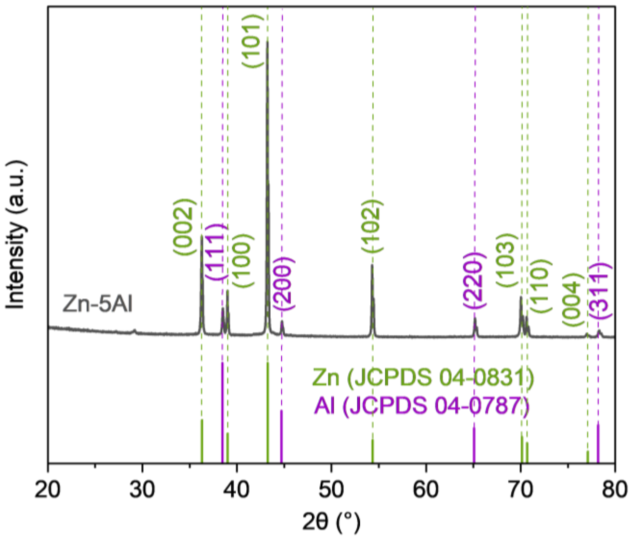

Figure S1. XRD pattern of eutectic Zn-5Al (wt%) precursor alloy.

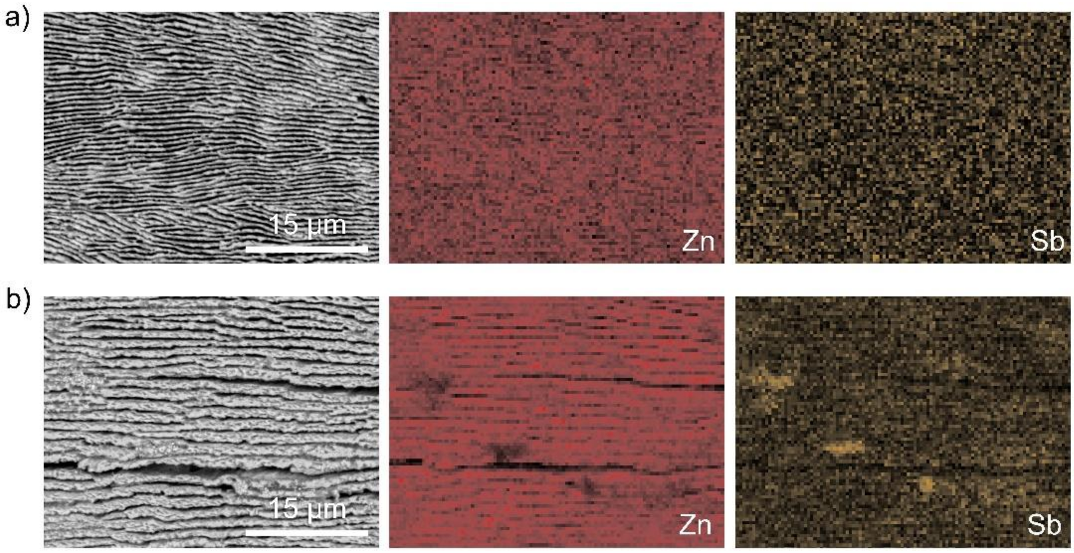

Figure S2. SEM images of a) Sb-pZn-310 and b) Sb-pZn-750 with the corresponding EDS elemental mappings of Zn and Sb.

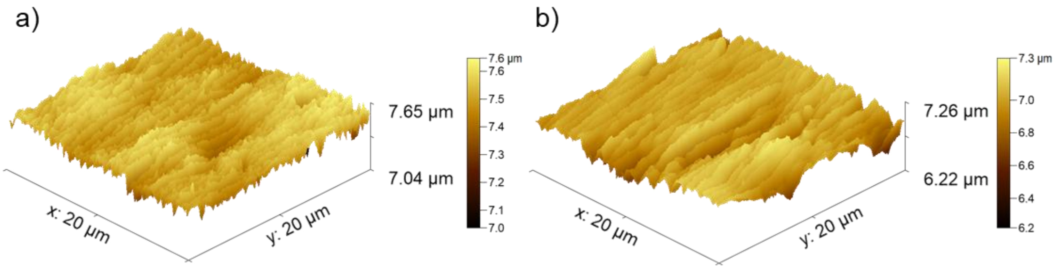

Figure S3. 3D topographic AFM images showing the surface elevation profiles of a) Sb-pZn-310 and b) Sb-pZn-750.

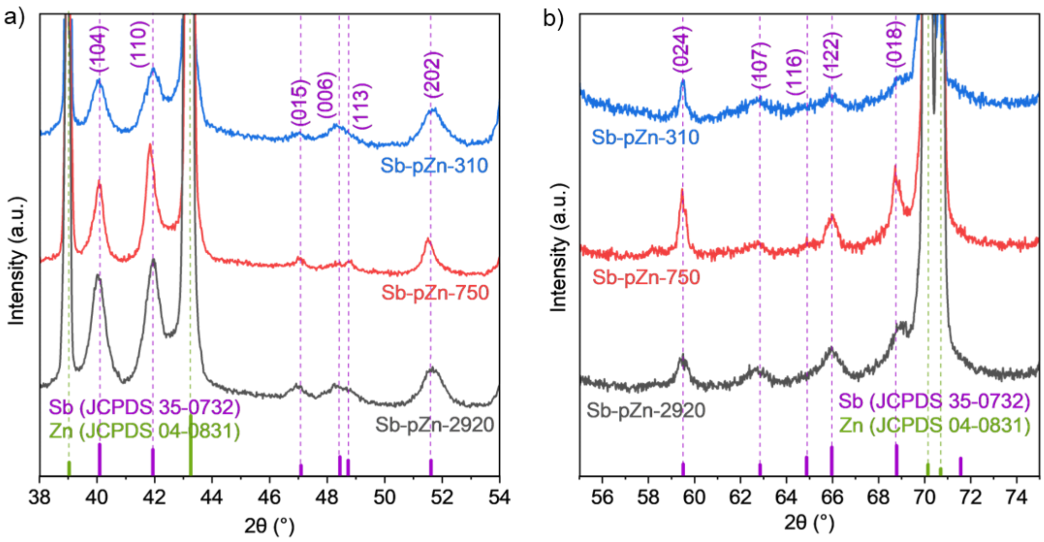

Figure S4. Enlarged views of XRD patterns showing the minor peaks of hexagonal Sb.

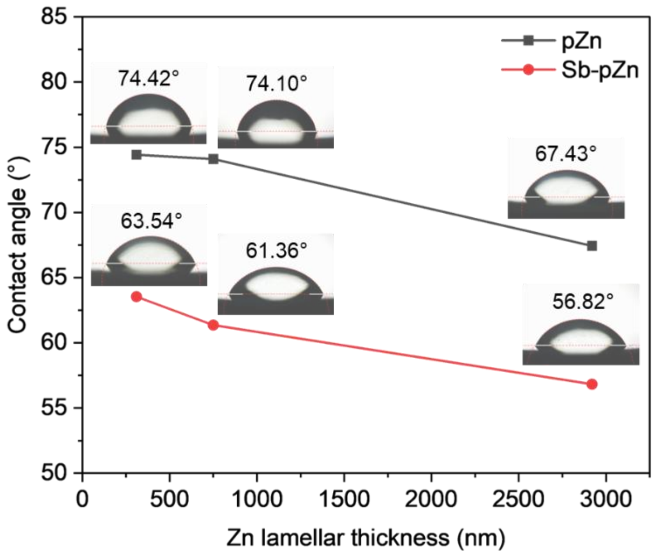

Figure S5. Comparison of Zn-ion electrolyte contact angle at different Zn lamellar thickness.

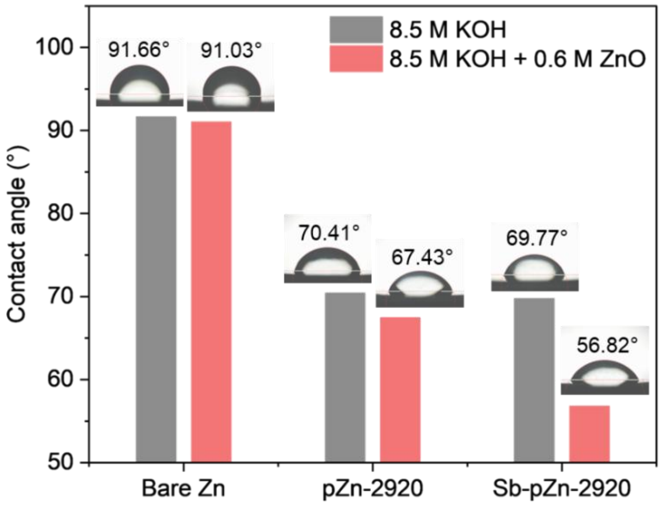

Figure S6. Comparison of electrolyte contact angle with and without Zn-ions on bare Zn, pZn-2920 and Sb-pZn-2920.

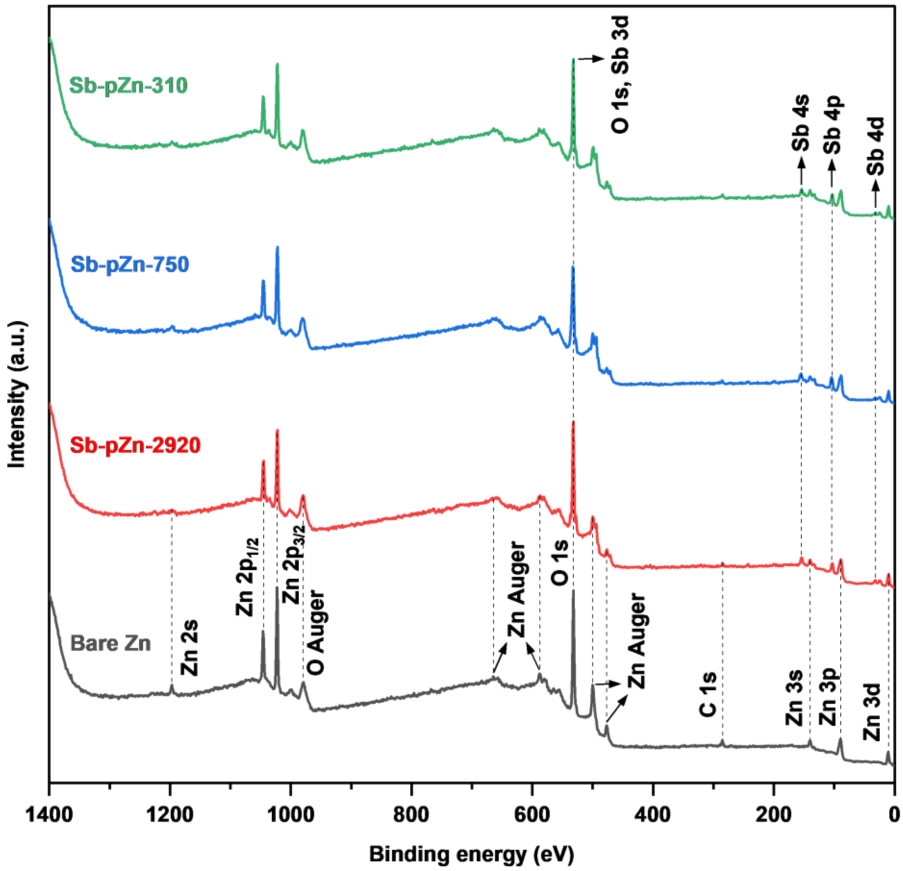

Figure S7. XPS survey scan of bare Zn, Sb-pZn-310, Sb-pZn-750 and Sb-pZn-2920.

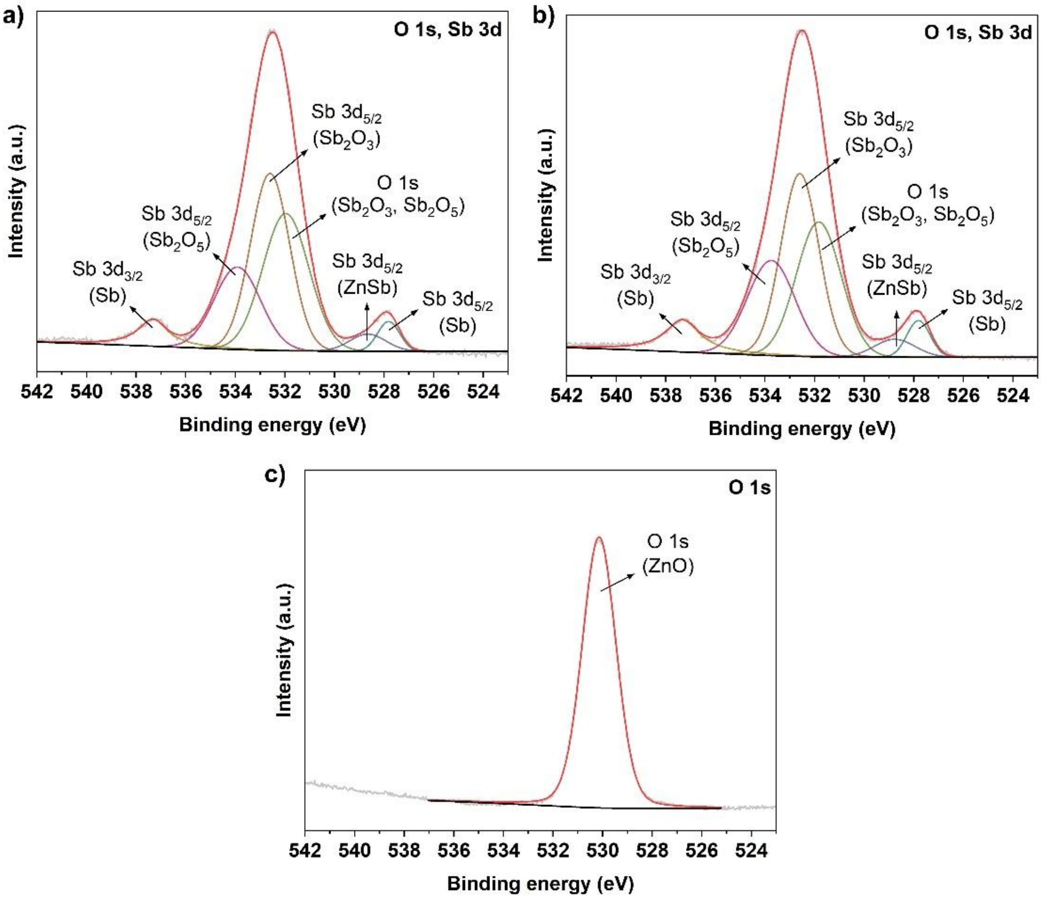

Figure S8. High-resolution O 1s and Sb 3d XPS spectra of a) Sb-pZn-310, b) Sb-pZn-750 and c) bare Zn.

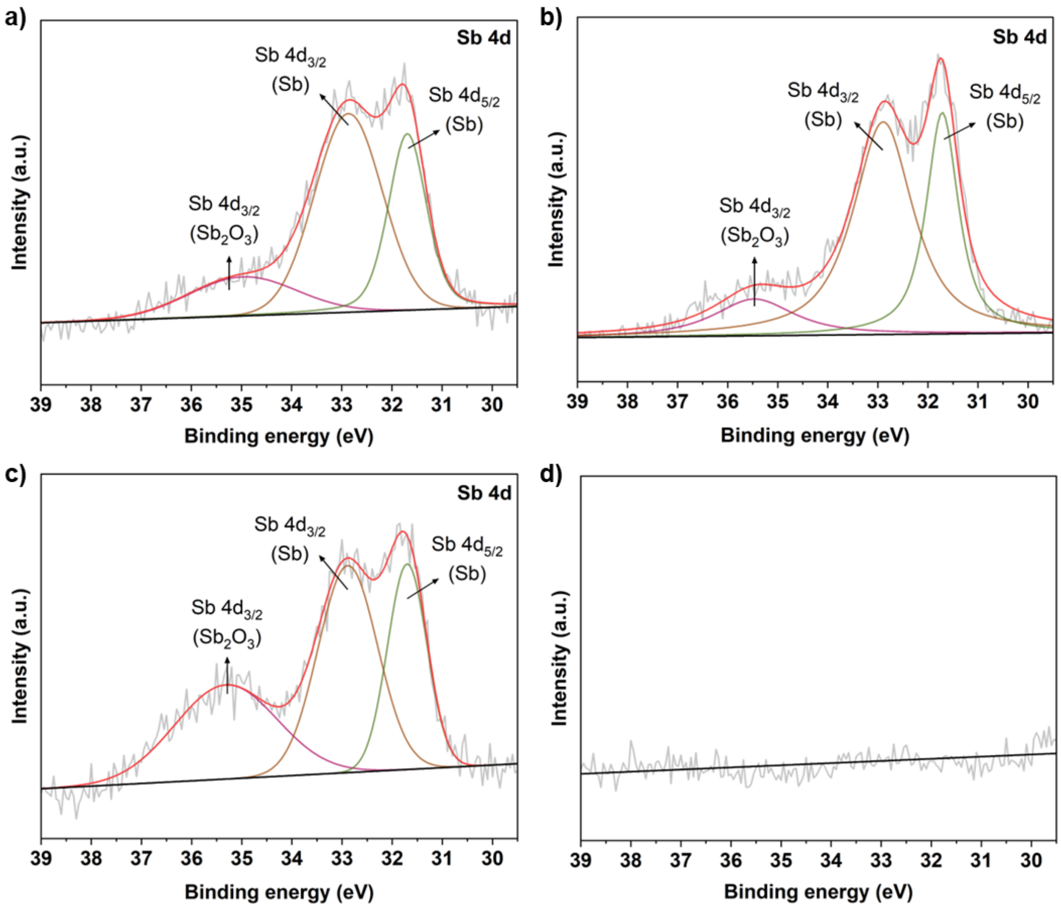

Figure S9. High-resolution Sb 4d XPS spectra of a) Sb-pZn-310, b) Sb-pZn-750, c) Sb-pZn-2920 and d) bare Zn.

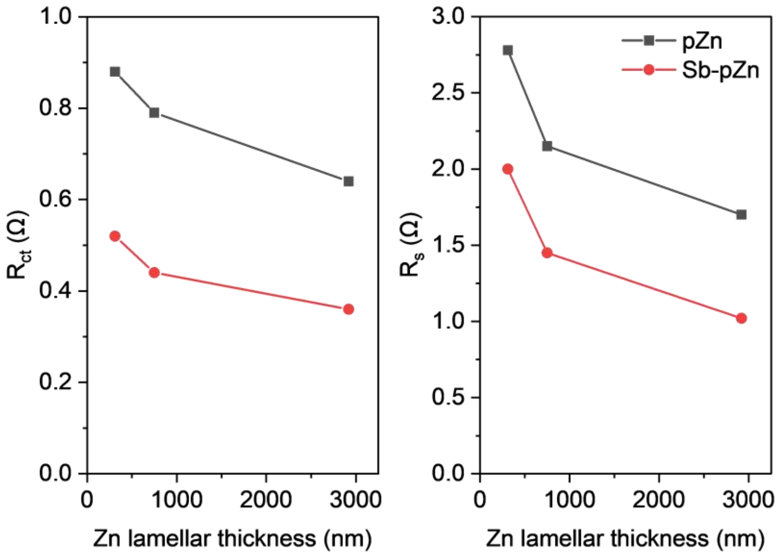

Figure S10. a) R_{ct} and b) R_s values of pZn and Sb-pZn as a function of Zn lamellar thickness.

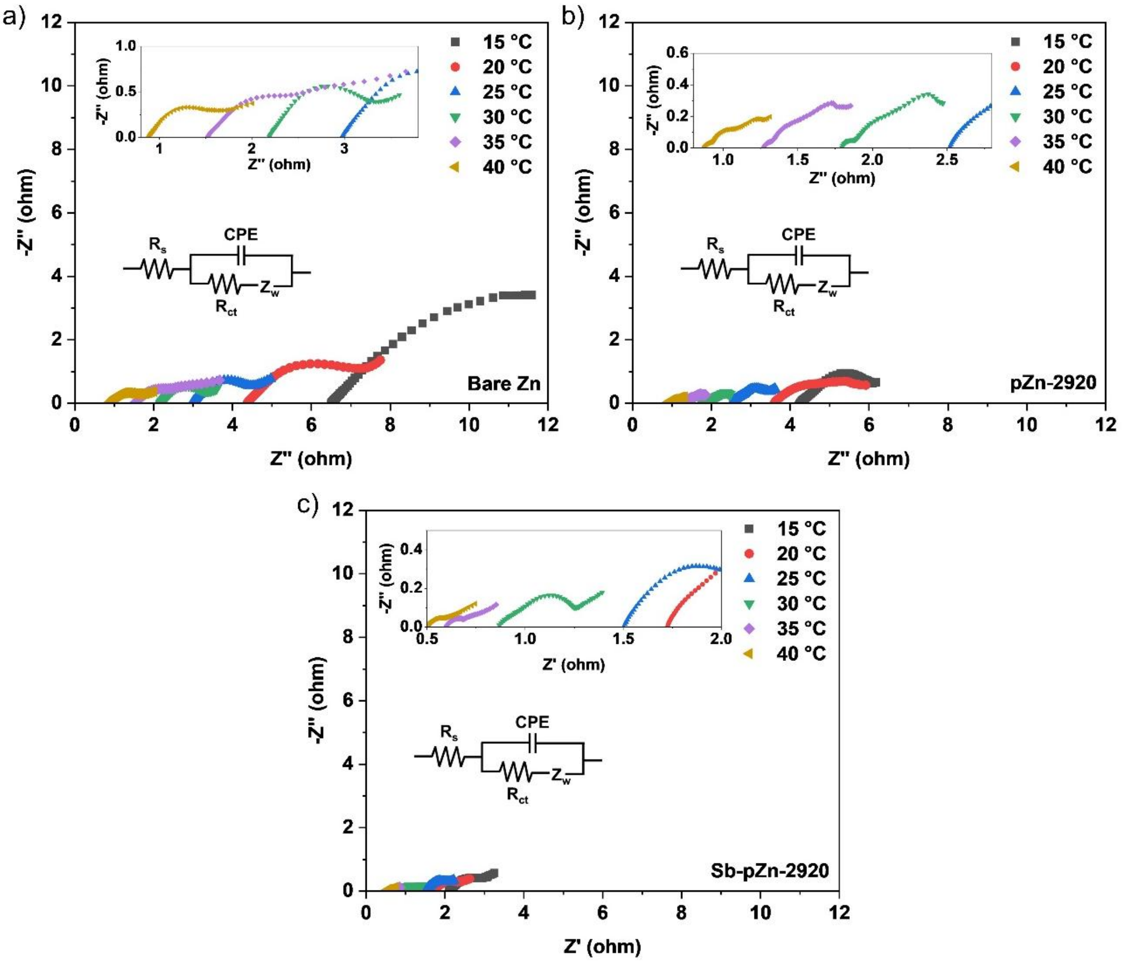

Figure S11. Nyquist plots of a) bare Zn, b) pZn-2920 and c) Sb-pZn-2920 symmetric cell at different temperatures.

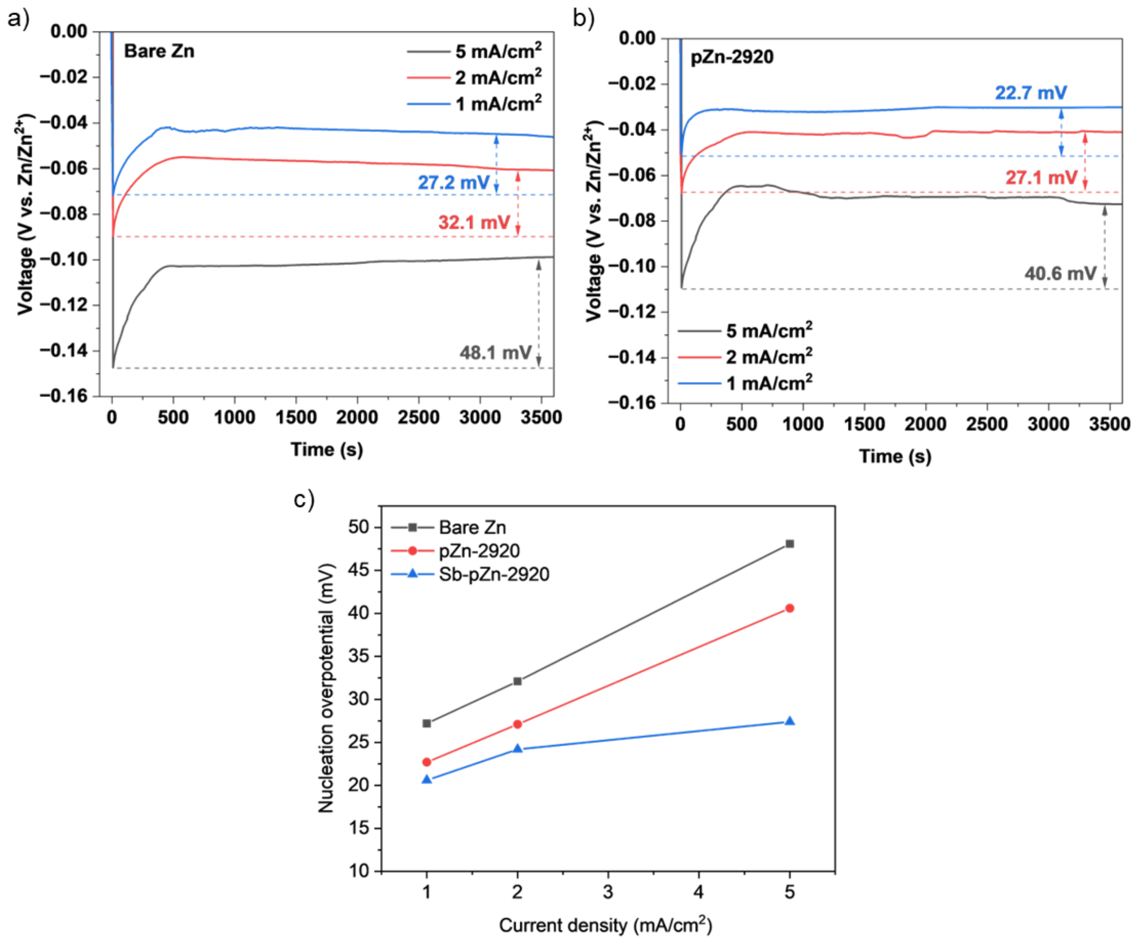

Figure S12. Nucleation overpotentials of a) bare Zn and b) pZn-2920 at current density from 1 to 5 mA cm^{-2}. c) Nucleation overpotentials as a function of current density.

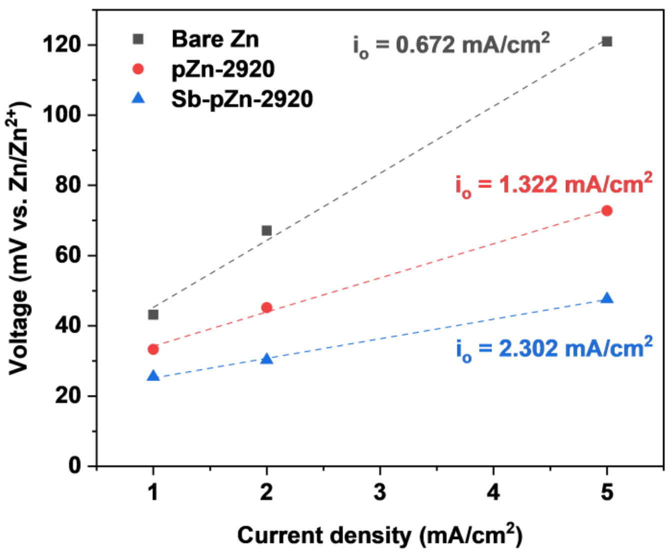

Figure S13. Exchange current densities of Sb-pZn-2920, pZn-2920 and bare Zn calculated according to rate performance.

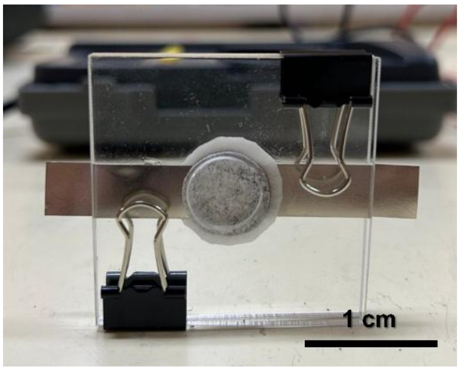

Figure S14. Photograph of the custom-made zinc-air battery cell assembled with Sb-pZn-2920 anode and air cathode.

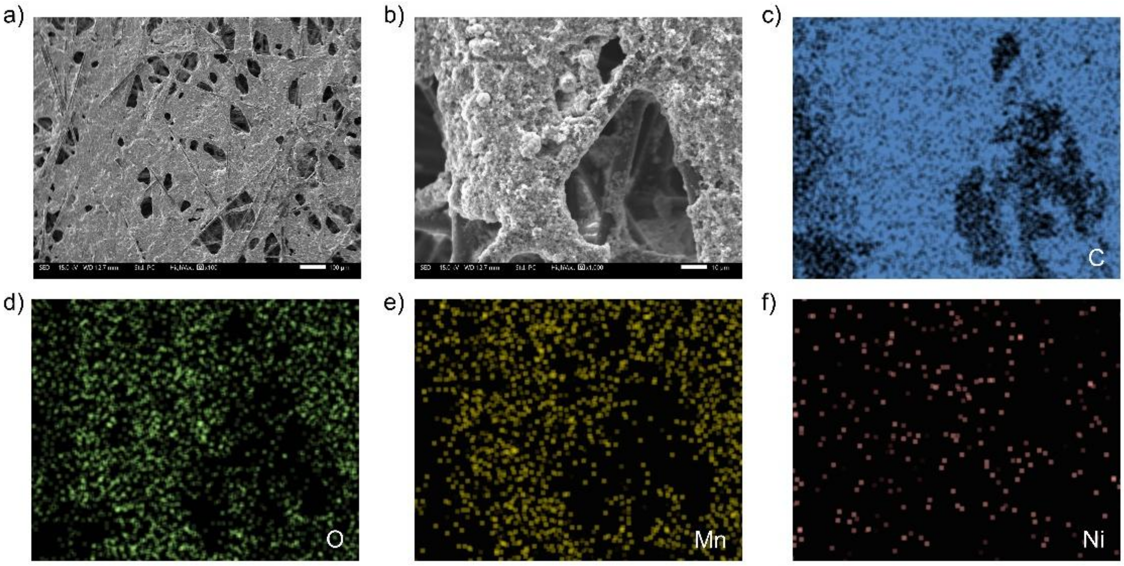

Figure S15. SEM images of air cathode and its corresponding EDS elemental mappings of C, O, Mn and Ni elements.

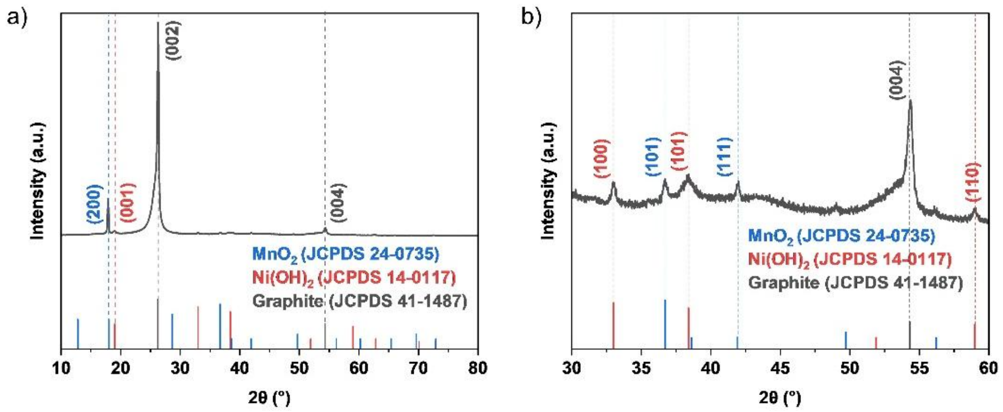

Figure S16. XRD patterns of air cathode.

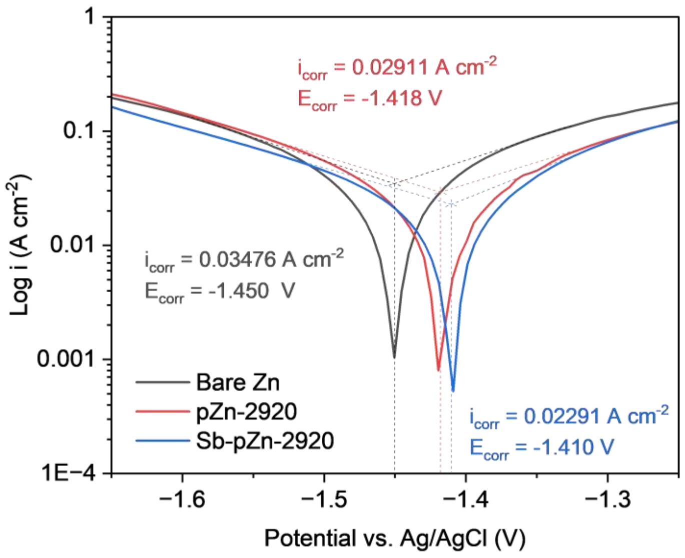

Figure S17. Tafel polarization curves of Sb-pZn-2920, pZn-2920 and bare Zn at scan rate of 1 mV s^{-1}.

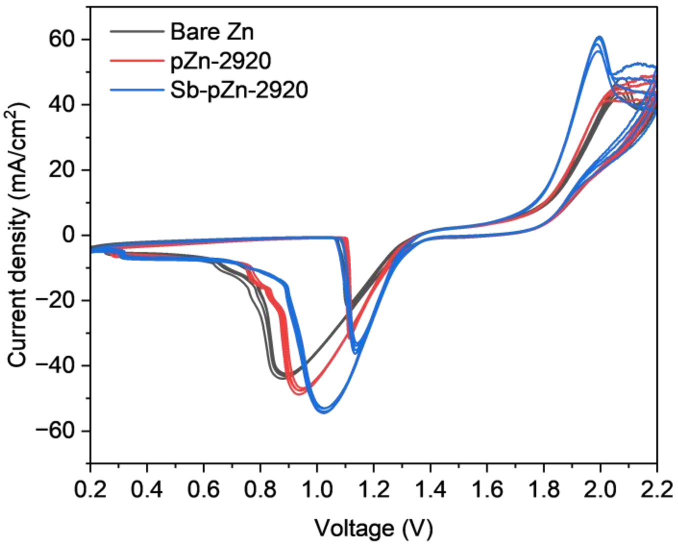

Figure S18. CV curves of Sb-pZn-2920||O_2, pZn-2920||O_2 and bare Zn||O_2 full cells at scan rate of 10 mV s^{-1}.

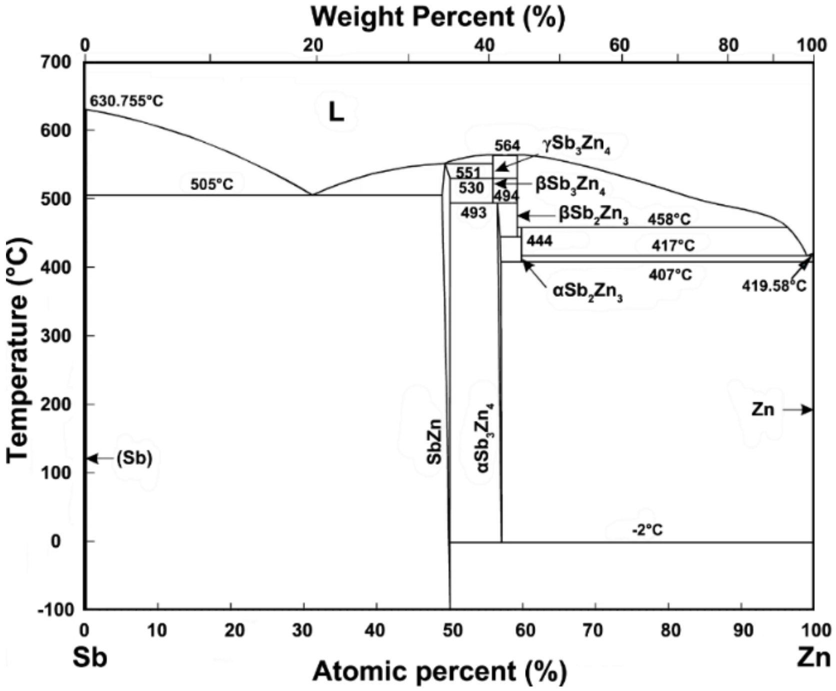

Figure S19. Zn-Sb binary phase diagram.

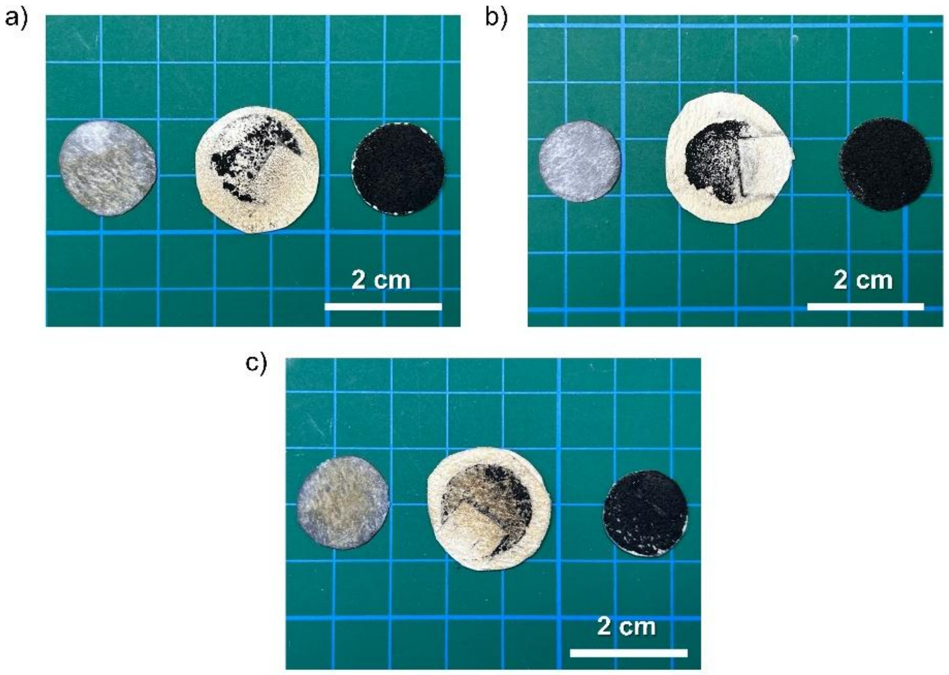

Figure S20. Disassembled anodes, separators and cathodes from a) Sb-pZn-2920||O_2, b) pZn-2920||O_2 and c) bare Zn||O_2 cells after cycling tests at 10 mA cm^{-2}.

[1] Sun W, Ma M, Zhu M, et al. Chemical Buffer Layer Enabled Highly Reversible Zn Anode for Deeply Discharging and Long-Life Zn–Air Battery. Small. 2022;18(9):2106604.
[2] Liu W, Li C, Li D, et al. Constructing zinc-tin alloy interface for highly stable alkaline zinc anode. Chinese Chemical Letters. 2025;36(7):110152.
[3] Wu W, Yang X, Wang K, et al. Inducing the Solid–Liquid Conversion of Zinc Metal Anode in Alkaline Electrolytes by a Complexing Agent. Advanced Functional Materials. 2022;32(45):2207397.
[4] Liu X, Wang H, Fan X, et al. Stabilizing zinc anodes for long-lifespan zinc–nickel battery through the in-situ construction of zincophilic interface layer. Energy Storage Materials. 2023;58:311-321.
[5] He J, Zhou W, Li J, et al. Bifunctional interface of metal–organic framework synergized with bismuth endows high stable zinc anode. Chemical Engineering Journal. 2024;485:149740.
[6] Peng Y, Lai C, Zhang M, et al. Zn–Sn alloy anode with repressible dendrite grown and meliorative corrosion resistance for Zn-air battery. Journal of Power Sources. 2022;526:231173.
[7] Fan X, Wang H, Liu X, et al. Functionalized Nanocomposite Gel Polymer Electrolyte with Strong Alkaline-Tolerance and High Zinc Anode Stability for Ultralong-Life Flexible Zinc–Air Batteries. Advanced Materials. 2023;35(7):2209290.
[8] Gang Y, Li X, Liu L, et al. Highly (002)-oriented ZnO in ZnO-N-C microflakes coating layer for stable zinc anode in zinc-air batteries. Electrochimica Acta. 2024;477:143816.
[9] Jiang P, Wang Y, Li Y, et al. Nitrogen-coordinated iron single atoms porous carbon as multi-functional artificial interface layer to enable self-purified zinc anode for rechargeable zinc-air batteries. Separation and Purification Technology. 2024;341:126813.
[10] Li L, Tsang YCA, Xiao D, et al. Phase-transition tailored nanoporous zinc metal electrodes for rechargeable alkaline zinc-nickel oxide hydroxide and zinc-air batteries. Nature Communications. 2022;13(1):2870.
[11] Liu P, Ling X, Zhong C, et al. Porous Zinc Anode Design for Zn-air Chemistry [Original Research]. Frontiers in Chemistry. 2019;Volume 7 - 2019.
[12] Zhang J, Li XL, Fan S, et al. 3D-printed functional electrodes towards Zn-Air batteries. Materials Today Energy. 2020;16:100407.
[13] Yu J, Chen F, Tang Q, et al. Ag-Modified Cu Foams as Three-Dimensional Anodes for Rechargeable Zinc–Air Batteries. ACS Applied Nano Materials. 2019;2(5):2679-2688.
[14] Hui X, Zhang P, Li J, et al. In Situ Integrating Highly Ionic Conductive LDH-Array@PVA Gel Electrolyte and MXene/Zn Anode for Dendrite-Free High-Performance Flexible Zn–Air Batteries. Advanced Energy Materials. 2022;12(34):2201393.
[15] Mei Z, Li H, Wang G, et al. Solvent-free and in situ synthesis of three-dimensional covalent organic frameworks thin films on Zn anodes for Zn–air batteries. Applied Surface Science. 2023;615:156324.
[16] Dilshad KAJ, Rabinal MK. Rationally Designed Zn-Anode and Co3O4-Cathode Nanoelectrocatalysts for an Efficient Zn–Air Battery. Energy & Fuels. 2021;35(15):12588-12598.
[17] Xiong J-F, Wang M-Y, Huang R-B, et al. Recent progress of <i>in-situ</i>/operando characterization approaches of zinc-air batteries. Chemical Synthesis. 2024;4(1):7.
